# Gestational hyperglycaemia impacts glucose control and insulin sensitivity in mouse offspring

**DOI:** 10.1038/s41598-025-91662-0

**Published:** 2025-02-28

**Authors:** K. Hribar, J. C. Fisher, D. Eichhorn, M. Smit, N. J. Kloosterhuis, B. M. Bakker, M. H. Oosterveer, J. K. Kruit, E. M. van der Beek

**Affiliations:** 1 Department of Paediatrics, University Medical Center Groningen, University of Groningen, Groningen, The Netherlands; 2 Department of Biochemistry, Stellenbosch University, Stellenbosch, South Africa; 3 Department of Laboratory Medicine, University Medical Center Groningen, University of Groningen, Groningen, The Netherlands; 4 The Central Animal Facility, University Medical Center Groningen, Groningen, The Netherlands; 5 Present Address: Nestlé Institute of Health Sciences, NestléResearch, Lausanne, Switzerland

**Keywords:** Metabolic diseases, Disease model

## Abstract

Gestational diabetes mellitus (GDM) predisposes offspring to the development of obesity and type 2 diabetes. While GDM is studied in the context of maternal obesity and insulin resistance, the consequences of GDM in lean, insulin sensitive women for offspring health are unclear. This preclinical study investigated whether GDM in lean dams characterized by reduced insulin secretion affects offspring metabolic health. Lean GDM was induced by short-term 60% high-fat diet and low-dose streptozotocin injections before mating in mice. The control dams received only high-fat diet (HF) or low-fat diet (LF). Glucose homeostasis was studied in chow-fed offspring. GDM resulted in decreased birth weight, that resolved at postnatal day 15 (PN15). At PN100, higher postprandial glucose responses were found in GDM offspring, while insulin secretion was lower in both GDM and HF offspring. Female GDM offspring showed lower endogenous glucose production and increased liver insulin sensitivity at PN100 compared to controls. No differences in metabolic parameters were observed at PN200 and PN300. Prenatal exposure to elevated maternal glucose levels without maternal obesity modestly affected glucose regulation in mouse offspring during early adulthood. Future studies should clarify if a less favourable postnatal diet may further challenge metabolic health in offspring of GDM dams.

**Supplementary Information:**

The online version contains supplementary material available at 10.1038/s41598-025-91662-0.

## Introduction

Type 2 diabetes (T2DM) is a multifactorial chronic disease affecting multiple organ functions involved in glucose homeostasis^1^. While genetic factors contribute to susceptibility to T2DM, they only account for a fraction of the overall risk^2^. Early developmental factors, like adverse intrauterine conditions such as maternal hyperglycaemia, play a significant role in T2DM susceptibility later in life^2,3^. Gestational diabetes mellitus (GDM) is the major cause of maternal hyperglycaemia, which is typically diagnosed in the late second or third trimester of pregnancy and differs from pre-existing diabetes because it develops over the course of gestation^4^. GDM is caused by a failure of insulin secretion and/or a relative insulin deficiency to compensate for insulin resistance during pregnancy resulting in a pregnancy related hyperglycaemia. GDM is a heterogenous disease with pathophysiological subtypes characterized by the degree of insulin resistance^4^. While efforts have focused on managing hyperglycaemia in pregnancy to reduce complications in mothers, addressing the consequences of the different subtypes for the offspring has received far less attention.

GDM has been linked to adverse long-term consequences in the offspring, such as a higher likelihood of developing obesity, glucose intolerance and T2DM in adulthood^5–9^. Early on, GDM influences foetal growth due to increased nutrient supply in combination with foetal hyperinsulinaemia^10^. This might be subtype specific as birthweight has been shown to be especially increased in offspring of women with insulin resistant driven GDM, but this has been more moderate or absent in offspring of women with normal insulin-sensitivity as well as impaired insulin secretion^11,12^. Childhood obesity risk is increased in offspring of GDM^13^, which is worsened in offspring of women with insulin resistant GDM^14^. Follow up studies from the offspring of the HAPO study showed that untreated moderate GDM is a significant risk factor for impaired glucose tolerance (IGT) in offspring at 10–14 years of age even after adjustment for offspring BMI^6^. Adjustment of IGT risk for maternal BMI did not change this relationship^6^, suggesting this is not GDM subtype specific. To study the mechanisms involved, preclinical studies have delved into the link between GDM and metabolic disturbances in offspring, particularly in obese and insulin-resistant models^15–18^. Maternal factors such as hyperglycaemia, insulin resistance, obesity, and dyslipidaemia have been identified as significant contributors to the adverse health outcomes in offspring^19^. Given the interconnected nature of these factors and the variation of these factors in the different GDM subtypes, it is crucial to determine whether hyperglycaemia as such, independent of obesity and insulin resistance, is a driver of the outcomes observed in the offspring.

Previously, we developed a lean, insulin sensitive GDM mouse model^20,21^. The double-hit model uses a high fat (HF) diet and streptozotocin (STZ) to induce transient gestation-specific lean hyperglycaemia. At the onset of pregnancy, these mice exhibit normal glucose levels, but as pregnancy progresses, dams develop glucose intolerance as a consequence of the impaired insulin secretion while insulin demands are increasing. These dams did not differ in weight from controls^22^, allowing us to focus on the effects of gestational glucose intolerance in the absence of maternal obesity and insulin resistance.

The aim of this study was to determine whether maternal GDM in absence of maternal obesity and insulin resistance affects offspring glucose homeostasis and tissue-specific insulin sensitivity. To this end, we used an oral glucose tolerance test (OGTT) and a newly established mixed meal tolerance test (MMTT) protocol, including using [U-¹³C₆]-glucose, combined with computational modelling^23^ to investigate the adaptive metabolic interplay to maintain glucose homeostasis.

## Materials and methods

### Animals and experimental setup

The study adhered to EU animal procedure guidelines (Directive 2010/(63/EU on the protection of animals used for scientific purposes). The Dutch Central Authority for Scientific Procedueres on Animal Central Committee for Animal Testing and the University of Groningen’s Institutional Animal Welfare Body approved the protocol. The study is reported in accordance with ARRIVE guidelines. Study design, maternal outcomes and diabetic status of the dams been reported previously^22^. In summary, female C57BL/6NTac mice (Taconic, Denmark) were pair housed until gestation and were fed either a 10% low-fat diet or a 60% high-fat diet, while male C57BL/6NTac mice (Taconic, Denmark) were fed a standard low fat chow diet. The dam treatments were LF + vehicle (LF), HF + vehicle (HF), and HF + STZ (HFSTZ). After four weeks of diet exposure, the mice received either 60 mg/kg STZ (S0130, Sigma-Aldrich) or vehicle for three consecutive days. Twelve days after the injection, vaginal smears were obtained daily at 3 PM to assess the estrous cycle and mice were bred as described previously^22^. Mice with normoglycemia under nonfasted conditions at GD0 (< 12 mM) were screened for GDM using an OGTT at GD15^22^. Mice with baseline levels > 12 mM were considered (pre)diabetic and excluded from further experiments. Based on the litter sizes of previous studies using this GDM model^20,21^, litters were standardized to 5 pups at PN2, three females and two males if possible. If this was not possible due to unfavourable female/male ratio, cross fostering was applied to ensure 5 pups per dam. Beginning at PN2, offspring were weighed weekly. After weaning at PN21, mice were pair housed on a chow diet (AB diet) until PN72, after which the remaining sibling was housed individually until PN310. The one surplus female from each litter was included in a separate study. At PN122 and PN310 of age, male and female offspring were placed in metabolic monitoring systems (Supplemental Materials). The offspring were sacrificed at either PN72 or PN310 after a 6 h fast (6 AM-12 PM in the light phase) in their home cage, followed by cardiac puncture and cervical dislocation under isoflurane anaesthesia. Organ samples were removed and snap-frozen in liquid nitrogen before storage at − 80 °C or fixed in 4% (wt/vol) formaldehyde in PBS.

### Magnetic resonance imaging

From PN42 body composition was measured monthly in male and female offspring via nuclear magnetic resonance imaging (LF900II, Bunker Optics, Billerica, MA). Conscious mice were inserted into the MRI chamber in a plastic cylinder, and lean mass, fat mass, and water were quantified. Body fat percentage was calculated using fat mass divided by total body weight.

### Oral glucose and meal tolerance tests with glucose tracer (tracer OGTT and MMTT)

In the PN310 cohort, glucose tolerance was assessed longitudinally using OGTT and MMTT on PN95/100, 195/200, and 295/300. On the day of the experiment, offspring were fasted for 6 h (6AM-12PM) followed by fasting blood glucose (FBG) measurement and collection of a small blood sample from the tail vein on filter paper (Satorius stedim TFN 180 g/m2). Stably labelled glucose [U-¹³C₆]-glucose (tracer) was included in the OGTT/MMTT assay to enable dissection of endogenous glucose production (EGP) and determine insulin sensitivity using mathematical modelling of tracer-based glucose kinetics^23,24^. During the OGTT or MMTT, a glucose bolus was delivered via oral gavage containing D-glucose (1 g/kg BW in a 200 g/L solution, of which 5% w/w was a tracer) or a mixed meal (1 g/kg BW in a 200 g/L solution, of which 5% w/w was a tracer; Table S1). Blood glucose (BG) measurements and separate blood spots for tracer analysis were obtained at eight time points (0, 5, 10, 20, 30, 45, 60, 90, and 120 min). Additional blood samples for insulin quantification were collected on filter paper by tail vein bleeding at six time points (0, 10, 30, 60, 90, and 120 min). Insulin measurements were performed using an Ultra-Sensitive Mouse Insulin ELISA Kit (Crystal Chem Cat. #90080), as described previously^22^. To correct for differences in sample volumes between the blood spots and plasma samples, the concentrations derived from the blood spots were multiplied by 1.28 ^25^.

### Stable-isotope enrichment analysis, EGP calculation and insulin sensitivity indices

The fractional distribution of [U-^12^C_6_]-glucose was determined using gas chromatography-quadrupole mass spectrometry (Agilent 9575 C Inert MSD, Agilent Technologies) as described previously^24^). Glucose was extracted from the dried blood spots and converted into a pentaacetate derivative. Positive chemical ionization with ammonia enabled monitoring of ions m/z 408–412 (corresponding to m_0_–m_4_ mass isotopologues), which were corrected for fractional distribution owing to the natural abundance of isotopes using multiple linear regression^26^.

**Fig. 1:**
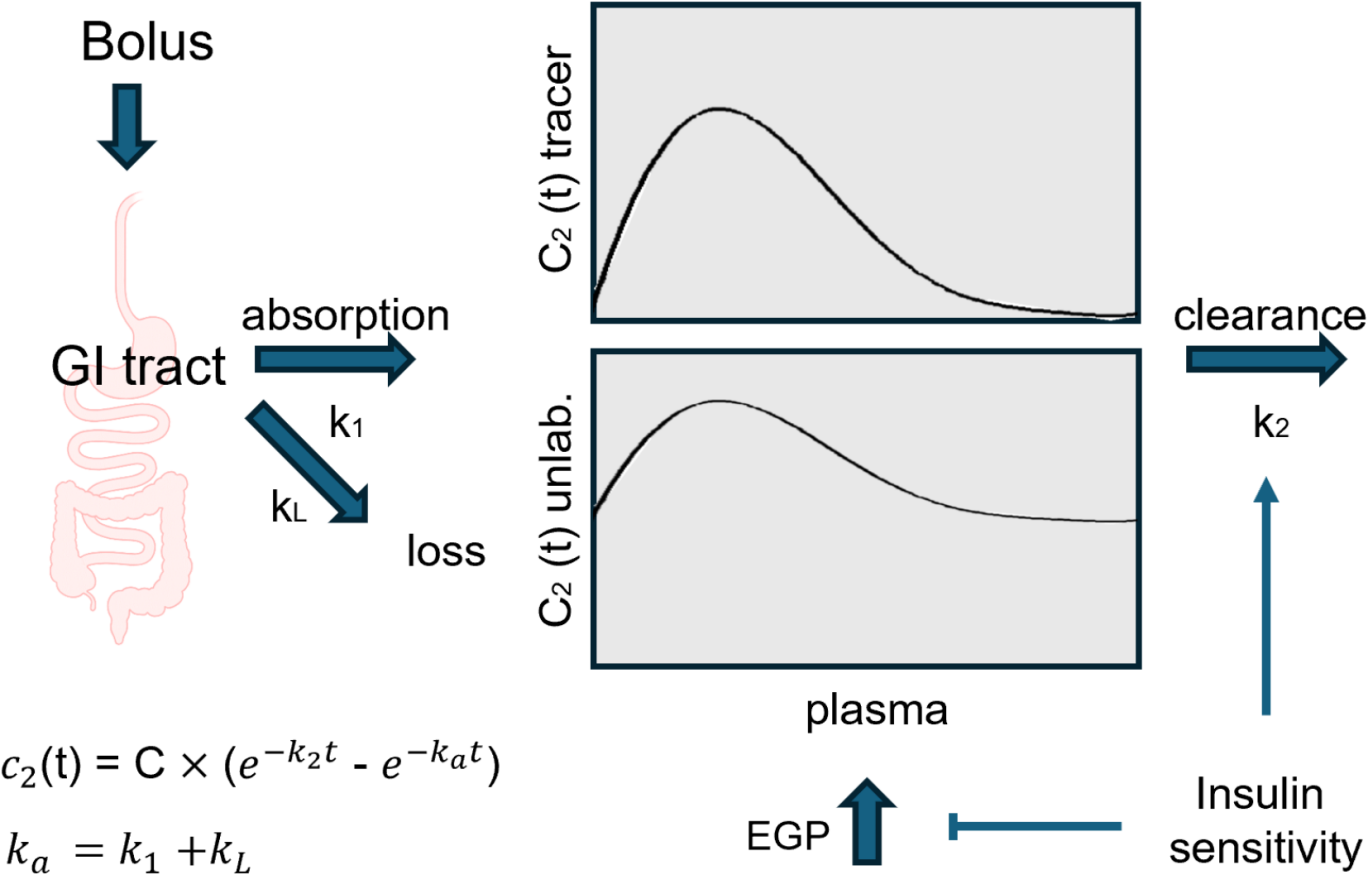
Compartment model for tracers and unlabelled glucose (Based on [20]). Gastrointestinal (GI) absorption flux into the plasma compartment, represented by the rate constant k_1_; loss, loss flux from the GI compartment, represented by the rate constant k_L_. Both tracer and unlabelled glucose in the plasma compartment can be cleared from the circulation, represented by the rate constant k_2_. Endogenous glucose production (EGP) by the liver feeds into the unlabelled glucose pool in the plasma. Insulin enhances the clearance of both tracer and glucose and inhibits the EGP. The apparent absorption constant k_a_ obtained from the tracer curves equals k_1_ + k_L_.

Kinetic parameters, endogenous glucose production (EGP), and insulin sensitivity indices were computed using the model described before^23^. Briefly, the compartment model depicted in Fig. 1 was used, with a gastrointestinal compartment (GI tract, compartment 1) and a blood plasma compartment (compartment 2). Here, labelled glucose pools (tracers) are represented by q1and q2, and corresponding unlabelled glucose pools by Q1 and Q2. The bioavailability F and apparent rate constant of appearance (*k*_*a*_) of the tracer equals: F = k_1_/(k_1_ + k_L_), k_a_ = k_1_ + k_L_. The time dependence of the plasma insulin levels was fitted to: $\:INS\left(t\right)=\:C\cdot\:\left({e}^{-{k}_{e}t}-{e}^{-{k}_{a}t}\right)$ in which INS is the fitted insulin concentration, and C, k_e_ and k_a_ are fitted constants. Average insulin over a specific time range ($\:{INS}_{{t}_{1}\to\:{t}_{2}}$) was computed as: $\:{INS}_{{t}_{1}\to\:{t}_{2}}=\frac{{AUC}_{{t}_{1}\to\:{t}_{2}}}{{t}_{2}-{t}_{1}}\:$ where, $\:{AUC}_{{t}_{1}\to\:{t}_{2}}\:$is the area under the fitted insulin curve from t_1_ to t_2_. The endogenous glucose production (EGP) was calculated from the times courses of labelled and unlabelled glucose, as described by^23^ with minor modifications. Following^23^ and inspired by^27^, peripheral insulin sensitivity (IS-P) was defined as follows: $\:{IS-P}_{T}=\frac{{k}_{2}}{{INS}_{0\to\:120}}\:$ in which k_2_ is the rate constant of peripheral glucose clearance (Fig. 1), as obtained from the fitting of the tracer time course (Fig. S1). The liver insulin sensitivity (IS-L) was defined as: $\:{IS-L}_{T}=\frac{\stackrel{-}{{EGP}_{{t}_{1}\to\:{t}_{2}}}\times\:\stackrel{-}{{INS}_{{t}_{1}\to\:{t}_{2}}}}{{EGP}_{{t}_{1}\to\:{t}_{2}}\times\:{INS}_{{t}_{1}\to\:{t}_{2}}}\:$as described previously in^23^, modified from^28^. HOMA-IR was calculated as previously described^24^. All calculations were conducted in Python using Jupyter Notebook (v7.0.1).

### Biochemical liver analysis

Liver lipids were extracted from 15% (w/v) liver homogenates in PBS, according to the method described by Bligh and Dyer^29^. Liver TGs (Roche) levels were analysed using commercially available kits.

### Software and analysis

Data are presented as individual data points as mean ± SEM with the dam as experimental unit. Off balance dam/offspring numbers are due to a failure to standardize to 3 females and 2 males in all cases. Sex differences (sex and sex x treatment group) were assessed using a two-way ANOVA. Between-group differences were tested using one-way ANOVA followed by Tukey’s multiple comparison test. All statistical analyses were performed using GraphPad Prism 10.2.3 software. Two-sided *p* < 0.05 was considered statistically significant.

The corrections for natural abundance of isotopes were performed using Excel 2023. Estimation of EGP rates, glucose clearance rates and insulin sensitivities were conducted in Python (version 3.12.0) using Jupyter Notebook (version 7.0.6). Parameters from the tracer kinetics and curves for the unlabelled glucose were estimated with the minimized function from the lmfit Python package (Levenberg-Marquardt method, nonlinear least squares). Data visualization and statistical analysis were conducted using Python (matplotlib, scipy) or GraphPad Prism (version 10.1.0; GraphPad Software, San Diego, CA, USA). Statistical analyses comparing the effects of sex and maternal exposure as well as their interactions were conducted using two-way ANOVA.

## Results

###  HF and GDM exposure during pregnancy result in decreased offspring body weight early in life

**Fig. 2:**
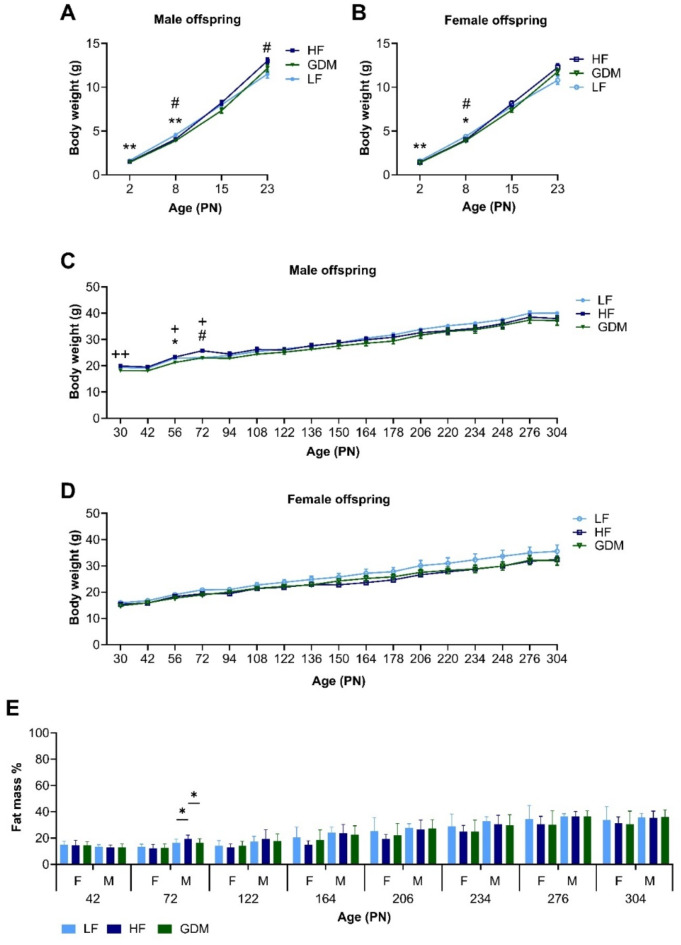
Offspring weight and body composition. Male (**A**) and female (**B**) offspring weights from PN2-23. Male (**C**) and female (**D**) offspring weights from PN30-304. (**E**) Fat mass of male and female offspring at mass from PN42-310. Offspring were maintained on a chow diet (CD) from PN21. Sample size until PN72 (offspring _dam_): male (LF:20_10_, HF:24_12_, GDM:26_13_) and female (LF:29_10_, HF:27 _12_, GDM:28_13_). Sample size after PN72: male (LF:10 _10_, HF:11_12_, GDM:13_13_), female (LF:9_10_, HF:11_12_, GDM:12_13_). Data are presented as the mean ± SEM, with each value from PN2 until PN72 representing the average of two siblings per sex from one litter, and after PN72 the measurement of the individual offspring from one litter. **A–C**: One-way ANOVA followed by Tukey’s multiple comparison test. *GDM vs. LF, ^+^GDM vs. HF, ^#^HF vs. LF. *p < 0.05, **p < 0.01, ***p < 0.001, ****p < 0.0001.

Breeding characteristics are given in Table 1. Maternal hyperglycaemia significantly reduced litter size at PN2 but had no effect on sex ratio (Table 1). Maternal hyperglycaemia resulted in decreased offspring body weight during early lactation in both sexes (LF vs. HF vs. GDM; PN2 male: 1.64 ± 0.04 vs. 1.50 ± 0.05 vs. 1.43 ± 0.04, female: 1.59 ± 0.04 vs. 1.43 ± 0.04 vs. 1.38 ± 0.04; PN8 male: 4.57 ± 0.08 vs. 4.07 ± 0.17 vs. 3.89 ± 0.17, female: 4.40 ± 0.07 vs. 3.99 ± 0.13 vs. 3.90 ± 0.16), which normalised by PN15 (Fig. 2A,B). Postweaning after a diet switch from high-fat milk to high-carbohydrate chow, GDM offspring showed similar body weight and adiposity compared to LF offspring. In addition, at adulthood tibia length was similar in all groups, indicating no differences in body length (Table S2). Offspring of dams that received a HF diet from pre-gestation onwards, showed decreased body weight during early lactation but following the transition to chow diet post-weaning, increased body weight was evident in males until PN72 compared to LF and GDM (Fig. 2C,D).

**Table 1:** Number of female breeders, number of pregnant mice, lost litters, final number of dams, litter size and litter sex. Lost litter = death of all pups and/or cannibalization between birth and PP2. Data on litter size and female sex are presented as mean ± sd. One-way ANOVA followed by Tukey’s multiple comparison test. *GDM vs. LF. ***p < 0.001. LF, low-fat diet; HF, high-fat diet; GDM, gestational diabetes mellitus.

	Mated female mice	Pregnant (%)	Lost litter (%)	Dams with litter included	Litter size	Female sex %, median (IQR)
Total	55	40	5	35		
LF	17	12 (75%)	3 (25%)	10	8.1 ± 2.0	42.2 (34.4–61.7)
HF	17	14 (82%)	2 (14%)	12	6.4 ± 1.4	35.4 (25.9–48.2)
GDM	21	14 (70%)	1 (7%)	13	5.0 ± 1.6***	50.0 (32.5–70.8)

Peak adiposity development is reached around postnatal day 72 (PN72) in mice. At this time point, the higher weight observed in HF males coincided with a noticeable increase in adiposity (Fig. 2E). The PN72 weight of sWAT (Table S2) and sWAT adipocyte surface area in HF males was significantly increased compared to LF and GDM (Fig. S2). Despite the increase in adiposity, no differences in plasma leptin, a marker of adiposity, were detected (Table S2). Maternal hyperglycaemia did not affect adiposity at any time. Both male and female GDM offspring showed adipose tissue levels similar to those of offspring from mothers kept on a LF diet (Fig. 2E).

To determine whether energy expenditure was affected in offspring of GDM dams, we performed indirect calorimetry over a 3-day period around PN122 and PN300. Respiratory exchange ratio, energy expenditure, glucose oxidation, food intake, and home cage activity were measured, but showed no significant differences between groups (Fig. S3). In addition, at PN300 no differences in body or organ weight, adiposity or plasma parameters were observed between groups (Fig. 2, Fig. S1 and Table S2). These results suggest that lean GDM only affects offspring body weight early in life.

### GDM exposure affects glucose and insulin responses in male and female mouse offspring in early adulthood

To determine if GDM exposure would affect glucose homeostasis in the offspring, we performed a detailed analysis of the glucose response to a glucose bolus (OGTT) or a mixed meal (MMTT). Systemic glucose tolerance in the offspring was assessed at PN100, PN200, and PN300. Fasting blood glucose levels remained unaffected across all groups in both sexes.

**Fig. 3:**
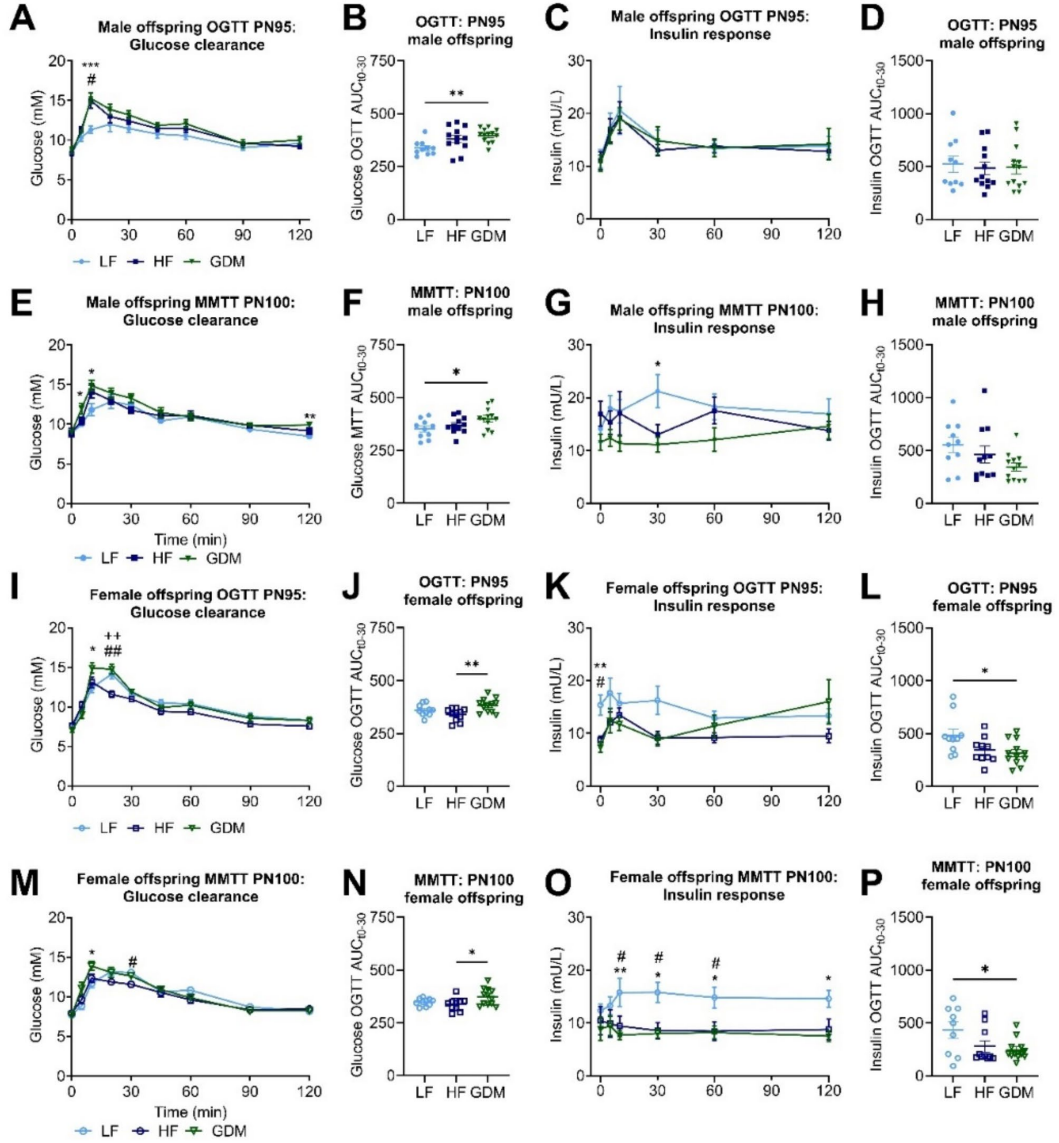
Glucose clearance and insulin response in male and female offspring during OGTT (PN95) and MMTT (PN100). Absolute glucose values during OGTT/MMTT in male (**A/E**) and female (**I/M**) offspring. AUC for glucose clearance induced by OGTT/MMTT (t0-30) in male (**B/F**) and female (**J/N**) offspring. Absolute insulin values during OGTT/MMTT in male (**C/G**) and female (**K/O**) offspring. AUC for insulin secretion induced by OGTT/MMTT (t0-30) in male (**D/H**) and female (**L/P**) offspring. Sample size (offspring _dam_): male (LF:10 _10_, HF:11 _12_, GDM:13 _13_), female (LF:9 _10_, HF:11 _12_, GDM:12 _13_). Data are presented as the mean ± SEM, with each value representing the average of the measurement of the individual offspring from one litter. **A**,** C**,** E**,** G**,** I**,** K**,** M**,** O**: Mixed effects 2-way ANOVA followed by Tukey’s multiple comparison test were used. **B**,** D**,** F**,** H**,** J**,** L**,** N**,** P**: Ordinary 1-way ANOVA analysis followed by Tukey’s multiple comparison test was used. * GDM vs. LF, + GDM vs. HF, # HF vs. LF. *p < 0.05, **p < 0.01, ***p < 0.001, ****p < 0.0001.

Around PN100, in the male offspring, both GDM and HF offspring exhibited higher OGTT peak glucose levels than LF offspring (Fig. 3A,B and Table S3). Female GDM offspring displayed earlier and higher OGTT blood peak glucose levels than LF and HF controls (Fig. 3I,J and Table S3). Similar differences in glucose response were noted using a meal tolerance test in GDM offspring compared to LF offspring (Fig. 3E,F,M,N and Table S3). Notably, insulin responses were comparable between groups during OGTT and MMTT for males (Fig. 3C,D,G,H and Table S3). The offspring of GDM females, however, exhibited a substantial reduction in fasting insulin levels at the onset of the OGTT and MMTT tests, as well as an attenuated insulin response (Fig. 3K,L,O,P and Table S3).

**Fig. 4:**
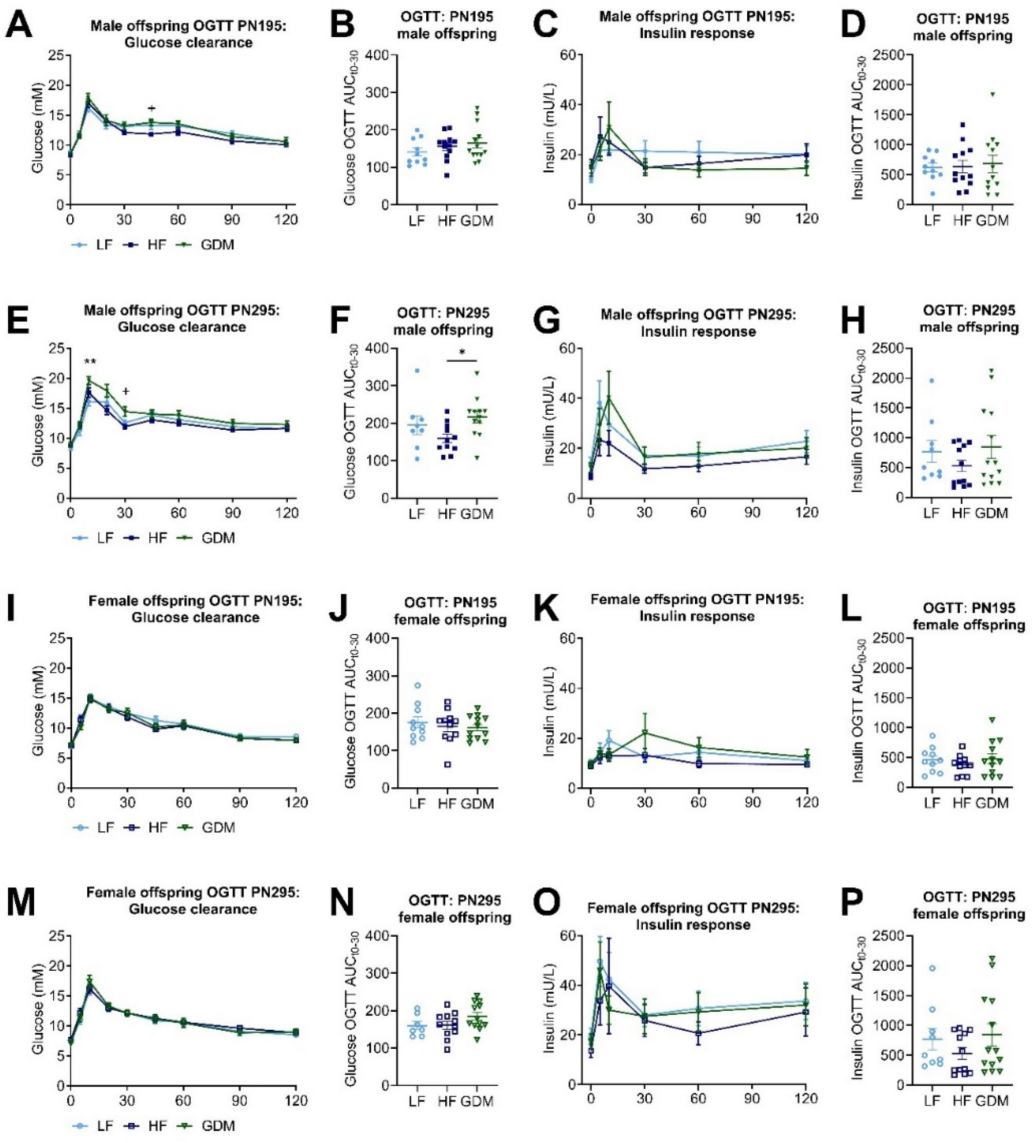
Glucose clearance and insulin response in male and female offspring during OGTT at PN195 and 295. Absolute glucose values during OGTT PN195/295 in male (**A/E**) and female (**I/M**) offspring. AUC for glucose clearance induced by OGTT PN195/295 (t0-30) in male (**B/F**) and female (**J/N**) offspring. Absolute insulin values during OGTT PN195/295 in male (**C/G**) and female (**K/O**) offspring. AUC for insulin secretion induced by OGTT PN195/295 (t0-30) in male (**D/H**) and female (**L/P**) offspring. Sample size (offspring _dam_): male (LF:10 _10_, HF:11 _12_, GDM:13 _13_), female (LF:9 _10_, HF:11 _12_, GDM:12 _13_). Data are presented as the mean ± SEM, with each value representing the average of the measurement of the individual offspring from one litter. **A**,** C**,** E**,** G**,** I**,** K**,** M**,** O**: Mixed effects 2-way ANOVA followed by Tukey’s multiple comparison test were used. **B**,** D**,** F**,** H**,** J**,** L**,** N**,** P**: Ordinary 1-way ANOVA analysis followed by Tukey’s multiple comparison test was used. * GDM vs. LF, + GDM vs. HF, # HF vs. LF. *p < 0.05, **p < 0.01, ***p < 0.001, ****p < 0.0001.

Around PN200, no changes were observed in glucose response or insulin release in either sex (Fig. 4 and Table S3). Around PN300, females again showed no changes (Fig. 4I–P and Table S3), but male GDM offspring showed higher OGTT glucose levels at timepoint 10 compared to LF offspring (Fig. 4E,F and Table S3). Nevertheless, no significant differences were observed in glucose clearance between the two groups during the MMTT (Fig. S4).

### Maternal GDM results in decreased endogenous glucose production only in female offspring

**Fig. 5:**
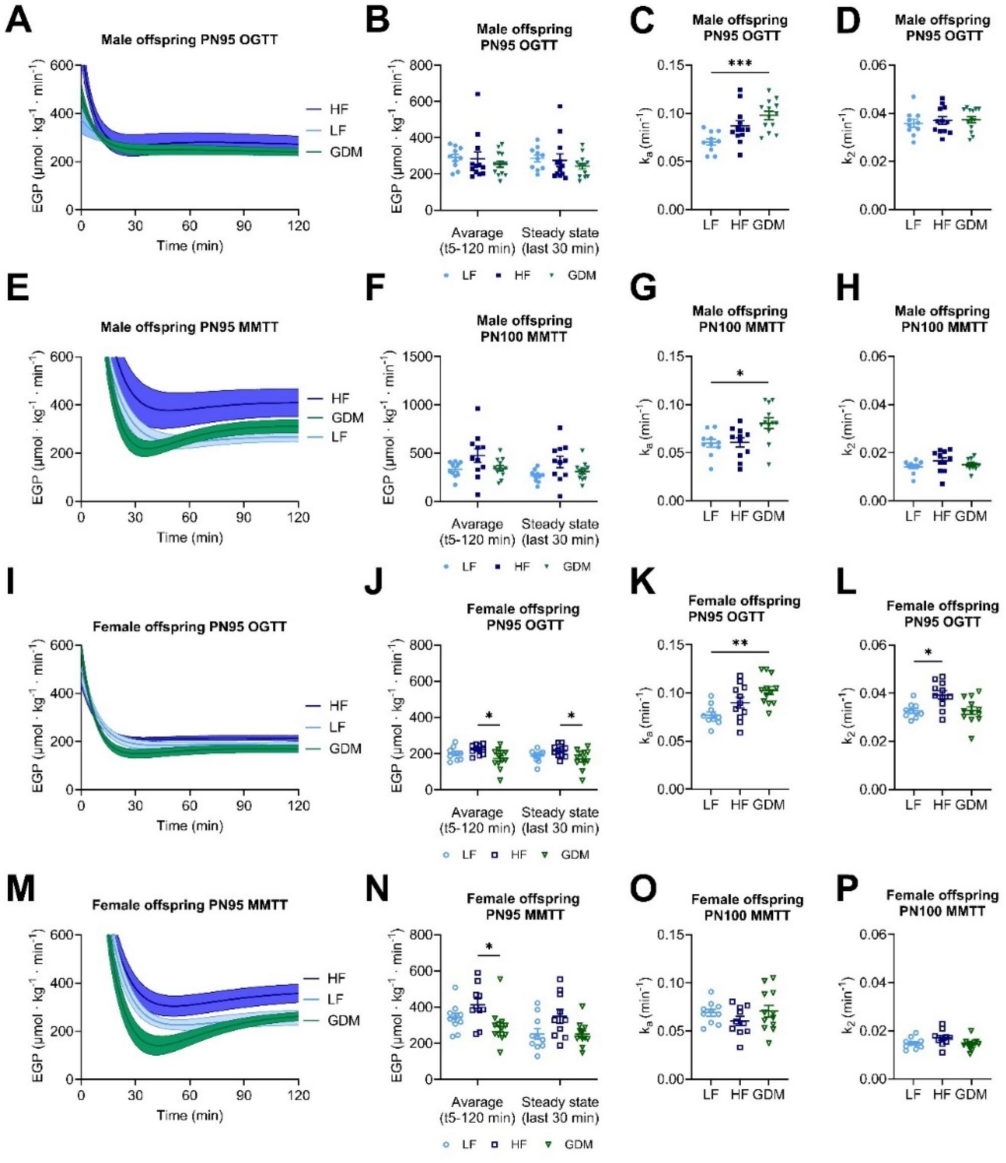
Endogenous glucose production and glucose clearance rates in male and female offspring during OGTT (PN95) and MMTT (PN100). Time course of EGP during OGTT/MMTT in male (**A/E**) and female (**I/M**) offspring. Time-averaged EGP values obtained from OGTT/MMTT and steady-state EGP values calculated from the curves in male (**B/F**) and female (**J/N**) offspring. The kinetic constants of OGTT/MMTT k_a_ in male (**C/G**) and female (**K/O**) and k_2_ in male (**D/H**) and female (**L/P**) from the curve fit. Sample size (offspring _dam_): male (LF:10 _10_, HF:11 _12_, GDM:13 _13_), female (LF:9 _10_, HF:11 _12_, GDM:12 _13_). **A-P**: Data are presented as the mean ± SEM, with each value representing the average measurement of the individual offspring from one litter. Ordinary 1-way ANOVA analysis followed by Tukey’s multiple comparison test was used. * GDM vs. LF, + GDM vs. HF, # HF vs. LF. *p < 0.05, **p < 0.01, ***p < 0.001, ****p < 0.0001.

Endogenous glucose production (EGP) by the liver regulates blood glucose levels and disturbed EGP contributes to the pathogenesis of diabetes. By the use of stable-isotope labelled glucose data of the OGTT and MTT, we determined the impact of maternal hyperglycaemia on offspring endogenous glucose production around PN100. After administration of the glucose bolus, the EGP decreased initially in all groups and both sexes, reaching a minimum between 10 and 30 min before reaching a new stationary level (Fig. 5A,I). After administration of the mixed-meal bolus, the EGP also resulted in an initial decrease in all groups and both sexes; however, reaching the minimum was delayed by 30 to 45 min compared to the OGTT (Fig. 5E,M). During the MMTT, maternal hyperglycaemia exposure led to a lower initial drop in EGP for both males and females compared to the OGTT. Steady-state EGP was calculated as the average of the last 30 min, and the overall average EGP was calculated over the entire period from 5 to 120 min. Exposure to maternal GDM decreased both the average EGP and steady-state EGP in the female offspring (Fig. 5N). Male mice showed similar EGP levels between groups (Fig. 5F). The apparent absorbance rate (k_a_) was higher in male GDM offspring, but not female GDM offspring (Fig. 5G,O).

During the OGTT, similar to the MTT data, the EGP was decreased in female GDM offspring, but not male GDM offspring (Fig. 5J,B). The apparent absorbance rate (k_a_) was higher in both female and male GDM offspring (Fig. 5C,K). The apparent rate constant k_2_ remained mostly unaltered during the OGTT and MMTT in both sexes across all groups, apart from an increase during the OGTT in the female offspring of HF dams (Fig. 5D,H,L,P). The constant k_2_ represents the fractional clearance rate of glucose from the plasma compartment and indicates that exposure to maternal GDM does not influence the “glucose effectiveness” of male and female offspring. These results lead us to the preliminary conclusion that elevated k_a_ contributes to increased glucose levels, while reduced EGP acts as a compensatory response in the opposite direction. It appears that k_a_ is the dominant factor contributing to the elevated glucose peak observed in the OGTT/ MMTT described in Fig. 3.

### Maternal GDM exposure affects offspring liver insulin sensitivity

**Fig. 6:**
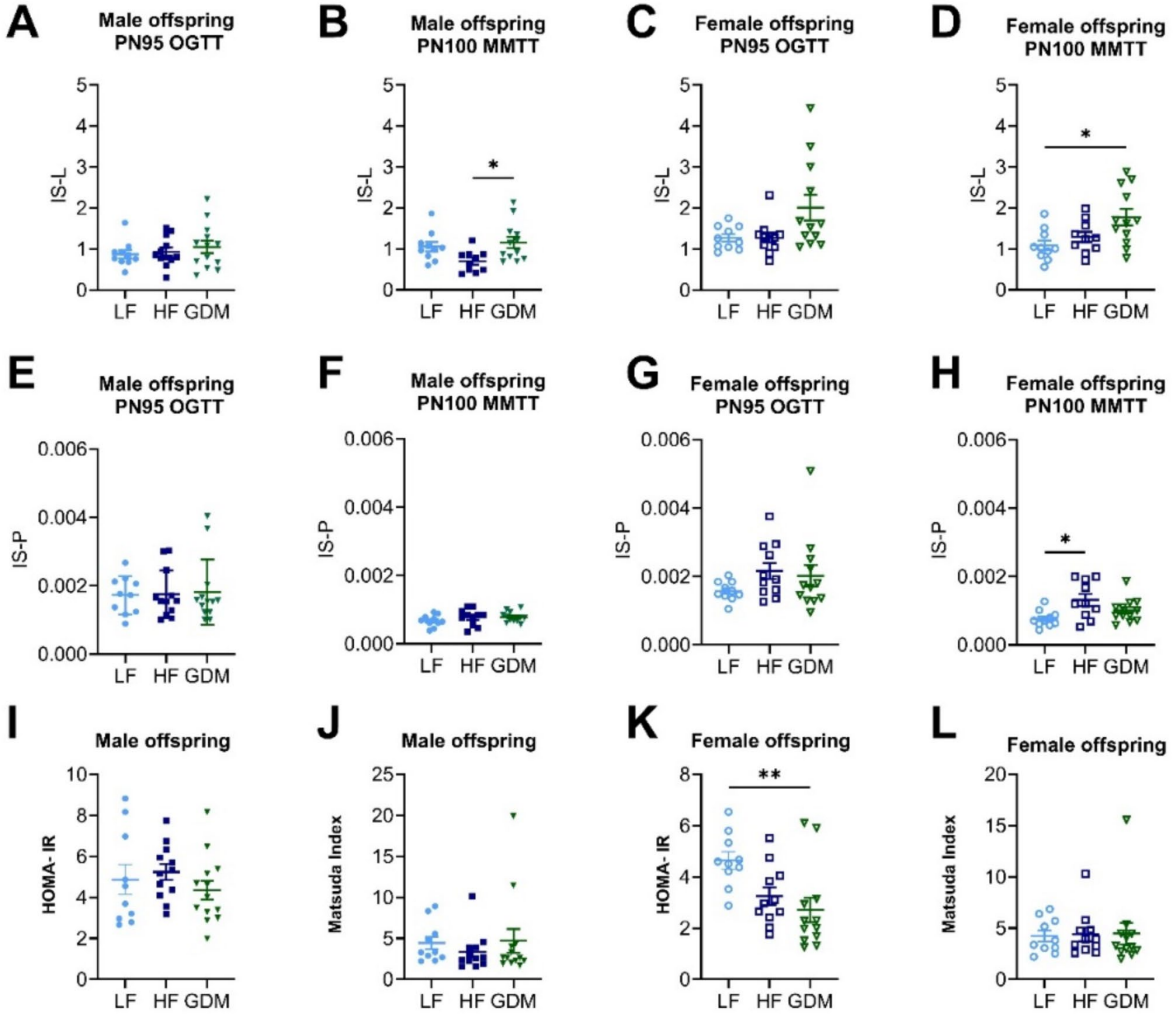
Peripheral and central insulin sensitivity. IS-L and IS-P for tracer OGTT (PN100)/MMTT (PN100), calculated according to the fractured equations in male (**A**,** B**,** E**,** F**) and female (**C**,** D**,** G**,** H**) offspring. Averaged Homa IR and Matsuda Index calculated from fasting glucose and fasting insulin levels during OGTT (PN100)/MMTT (PN100) in male (**I**,** J**) and female (**K**,** L**) offspring. Sample size (offspring _dam_): male (LF:10 _10_, HF:11 _12_, GDM:13 _13_), female (LF:9 _10_, HF:11 _12_, GDM:12 _13_). **A–L**: Data are presented as the mean ± SEM, with each value representing the average measurement of the individual offspring from one litter. Ordinary 1-way ANOVA analysis followed by Tukey’s multiple comparison test was used. * GDM vs. LF, + GDM vs. HF, # HF vs. LF. *p < 0.05, **p < 0.01, ***p < 0.001, ****p < 0.0001.

As hepatic glucose production is regulated by insulin, we calculated tissue-specific insulin sensitivity using the stable-isotope labelled glucose tracers. Although liver-specific insulin sensitivity (IS-L) during the OGTT was similar in all groups, the MMTT assay revealed a modest significant increase in IS-L during the MMTT in both sexes in GDM offspring (Fig. 6A–D). No significant differences were observed in peripheral insulin sensitivity (IS-P) in GDM offspring (Fig. 6E–H). HOMA-IR and Matsuda index, surrogate measurements of whole-body insulin sensitivity, were unaltered in male mice (Fig. 6I,J), but small changes in insulin sensitivity were noticed in female mice (Fig. 6K,L), reflected by the decrease in HOMA-IR in the GDM female offspring (Fig. 6K). Liver lipids influence hepatic insulin sensitivity and may contribute to the development of diabetes. Remarkably, both male and female offspring of GDM dams exhibited a significant reduction in liver triglycerides at PN72 compared to LF control offspring (LF vs. HF vs. GDM; PN72 male: 32.0 ± 3.6 vs. 23.4 ± 1.6 vs. 22.9 ± 1.3, GDM vs. LF *p* < 0.05, female: 33.8 ± 3.6 vs. 29.6 ± 1.6 vs. 21.4 ± 1.4 µmol/g liver, GDM vs. LF *p* < 0.05). Aging increased liver triglyceride levels, but there was no discernible impact of early life exposure to maternal GDM or HF diet on liver lipid accumulation in the offspring at PN300 (LF vs. HF vs. GDM; PN300 male: 55.8 ± 5.0 vs. 62.5 ± 8.9 vs. 59.8 ± 7.1, female: 45.7 ± 4.9 vs. 39.8 ± 2.5 vs. 41.2 ± 7.3 µmol/g liver).

## Discussion

In this study we determined the impact of impaired insulin secretion-driven GDM on glucose and insulin responses in adult male and female offspring using tracer-based OGTT and MMTT challenges. We found that in utero exposure to maternal hyperglycaemia in the absence of maternal obesity and insulin resistance affected glucose regulation and insulin sensitivity in offspring during early adulthood, despite consumption of a healthy diet since weaning. The changes in glucose regulation that may indicate an increased susceptibility to metabolic diseases, however, did not persist over time.

GDM is an important risk factor for fetal overgrowth and subsequently increased rates of large for gestational age (LGA) and macrosomia^4^, that can be reduced with GDM treatment^30,31^. Increased maternal insulin resistance plays a role as women with GDM driven by insulin resistance specifically show increased birthweight despite treatment^11,12^. Analysis of the HAPO study with moderate GDM cases without treatment, however, also found an increased LGA risk in the group of women with an insulin secretion driven GDM subtype^32^. Maternal hyperglycaemia is associated with hyperinsulemia in the fetus and can be observed as early as 14–16 weeks of gestation^33^. Early fetal abdominal overgrowth is present prior to diagnosis of GDM^34^. Preclinical studies, however, often show decreased birth weight of offspring of hyperglycemic dams^35,36^. In agreement with this, in this study offspring exposed to hyperglycaemia prenatally showed significantly lower birth weight followed by normalization post-weaning. One of the reasons behind this discrepancy in the association between GDM and birth weight might be the development differences between species. Whereas humans are born with a substantial amount of adipose tissue, mice are born with limited fat mass and adipose tissue development occurs mostly postnatally. Body weight of GDM offspring was normalised by PN15, indicating that GDM offspring showed catch up growth. Interestingly, fetal growth restriction followed by postnatal early catch-up growth is associated with increased risk of metabolic diseases in humans^37^.

GDM is associated with increased risk for type 2 diabetes in offspring^5,8,9^. The increased risk can partly be explained by the increased adiposity and BMI in offspring of GDM pregnancies^7,8^. However, glucose homeostasis was affected at 10–14 years of age in children of GDM mothers showing increased incidence of impaired glucose tolerance and insulin resistance even after adjustment for offspring BMI^6^. In our study, looking at the offspring of lean insulin sensitive GDM dams, we found changed glucose homeostasis around PN100, despite normal body weight and adiposity. Although the effect size is moderate and slightly more pronounced in the female offspring, one needs to keep in mind that the mice were metabolically unchallenged receiving a healthy chow diet from weaning onwards and were group housed. Future studies using metabolically challenging conditions should address the question whether the effect of GDM on offspring metabolic health can be affected by subsequent challenges, just like being described for preclinical studies using more insulin-resistant GDM models^15,16^.

The use of stably labelled glucose during the OGTT and the MTT enabled us to dissect the contribution of the EGP and glucose clearance by peripheral tissues^23,24^ and study the glucose homeostasis. We observed a perturbed glucose response in young adult GDM male and female offspring that showed distinct differences between the two challenges used, e.g. OGTT and MMTT. Although the OGTT provides useful information about glucose tolerance, it does not mimic a physiological postprandial response^38^. Metabolic feedback to a mixed meal is a better indication of beta-cell function under normal daily life conditions compared to the standard OGTT because the mixed meal contains proteins and fatty acids, which are components that can stimulate insulin secretion^38,39^. For instance, gastric emptying happens faster after the OGTT compared with a mixed meal because of readily available glucose, which leads to its fast release to the duodenum and the portal venous circulation. In addition, the protein and fat content present in the mixed meals delay gastrointestinal glucose absorption, which leads to lower profiles of postprandial glucose and different insulin secretion profiles^40,41^. In light of this, we carried out a MMTT, next to the standard OGTT. We observed a more pronounced effect on offspring insulin secretion profiles during MMTT compared to OGTT. The male and female offspring of mothers who consumed a HF diet during pregnancy overall displayed reduced insulin responses, but this effect was only significant in the offspring additionally exposed to maternal hyperglycaemia. This observation underscores the crucial role that maternal hyperglycaemia plays in programming offspring metabolic responses.

The early insulin release after ingestion of nutrients seems to play a critical role in the maintenance of glucose homeostasis^42^. A prompt release of insulin is necessary to restrain postprandial glucose excursions, through a prompt inhibition of EGP, a physiological glucose disposition and, finally, in reducing a more sustained secretory stress on the beta cell^42–44^. We examined the effect of maternal hyperglycaemia on EGP in offspring by utilizing a preexisting computational model^23^ to analyse the tracer data from the OGTT and MMTT. In GDM offspring, we observe lower EGP, which may be a beneficial adaptation in early life. However, while this adaptation might offer short-term physiological benefits, it could indicate adaptations in pancreas and liver function that may become a challenge longer term or after additional exposures/challenges. The low insulin levels suggest that the reduced response could be the result of decreased pancreas responsivity, potentially due to the effect of maternal hyperglycaemia on the developing pancreas. During pregnancy, the foetus is subjected to heightened levels of maternal glucose, which can cause changes in the development of the pancreas, such as reduced beta cell mass and impaired islet function^45^. These alterations may explain reduced insulin secretion and disrupted glucose metabolism as we observed in our study and should be investigated in further studies.

Previous studies examining the effects of maternal hyperglycaemia on developmental programming and offspring development have identified distinct outcomes for males and females^46,47^. Although in our study both sexes exhibited disrupted glucose and insulin metabolism in early adulthood, alterations in insulin levels at PN100 were specifically observed only in females. Furthermore, the effect of liver insulin sensitivity was more pronounced in female offspring. This aligns with prior research suggesting that females may be more susceptible to programming influencing glucose homeostasis^48^, whereas males seem more susceptible to changes in adiposity and body weight^49^. Whether this is influenced by GDM subtype is unclear.

In conclusion, our findings show a link between maternal GDM, in absence of insulin resistance and obesity, and metabolic outcomes in offspring. We show that combining MMTT with a stable glucose tracer compared to the standard OGTT provides a means to detect more subtle changes in insulin sensitivity by providing precise insulin secretion profiles that mimic the physiological postprandial metabolism. The observed lower EGP may beneficially adapt offspring to in utero hyperglycaemia. Nonetheless, the main conclusions regarding longer term outcomes remain consistent for both OGTT and MMTT, enhancing the robustness of our findings. In our study, prenatal exposure to elevated maternal glucose levels during pregnancy induced a modest adaptation in glucose metabolism in mouse offspring in early adult life. Future research is needed to further explore the mechanisms behind these early alterations and to determine whether long-term metabolic consequences may arise under less favourable dietary conditions.

## Electronic supplementary material

Below is the link to the electronic supplementary material.

Supplementary Material 1

## Data Availability

Data described in the manuscript can be made available upon reasonable request by contacting J.K Kruit (j.k.kruit@umcg.nl).

## References

[1] Chatterjee, S., Khunti, K. & Davies, M. J. Type 2 diabetes. *Lancet* **389**, 2239–2251 (2017). 28190580 10.1016/S0140-6736(17)30058-2

[2] Zheng, Y., Ley, S. H. & Hu, F. B. Global aetiology and epidemiology of type 2 diabetes mellitus and its complications. *Nat. Rev. Endocrinol.* **14**, 88–98 (2018). 29219149 10.1038/nrendo.2017.151

[3] Berends, L. M. & Ozanne, S. E. Early determinants of type-2 diabetes. *Best Pr Res. Clin. Endocrinol. Metab.* **26**, 569–580 (2012). 10.1016/j.beem.2012.03.002 22980041

[4] Hivert, M. F. et al. Pathophysiology from preconception, during pregnancy, and beyond. *Lancet*10.1016/s0140-6736(24)00827-4 (2024). 38909619 10.1016/S0140-6736(24)00827-4

[5] Feig, D. S. et al. Long-term neurobehavioral and metabolic outcomes in offspring of mothers with diabetes during pregnancy: A large, Population-Based cohort study in Ontario, Canada. *Diabetes Care*. 10.2337/dc24-0108 (2024). 38820461 10.2337/dc24-0108

[6] Lowe, W. L. et al. Hyperglycemia and adverse pregnancy outcome Follow-up study (HAPO FUS): Maternal gestational diabetes mellitus and childhood glucose metabolism. *Diabetes Care*. **42**, 372–380 (2019). 30655380 10.2337/dc18-1646 PMC6385693

[7] Bendor, C. D. et al. Glucose intolerance in pregnancy and offspring obesity in late adolescence. *Diabetes Care*. **45**, 1540–1548 (2022). 35670776 10.2337/dc21-2634

[8] Kaseva, N. et al. Gestational diabetes but not prepregnancy overweight predicts for cardiometabolic markers in offspring Twenty years later. *J. Clin. Endocrinol. Metab.* **104**, 2785–2795 (2019). 30835282 10.1210/jc.2018-02743

[9] Clausen, T. D. et al. High prevalence of type 2 diabetes and Pre-Diabetes in adult offspring of women with gestational diabetes mellitus or type 1 diabetes. *Diabetes Care*. **31**, 340–346 (2008). 18000174 10.2337/dc07-1596

[10] Meek, C. L. An unwelcome inheritance: childhood obesity after diabetes in pregnancy. *Diabetologia* 1–10 (2023). 10.1007/s00125-023-05965-w 10.1007/s00125-023-05965-w PMC10541526 37442824

[11] Selen, D. J. et al. Physiological subtypes of gestational glucose intolerance and risk of adverse pregnancy outcomes. *Am. J. Obstet. Gynecol.*** 226**, 241.e1-241.e14 (2022). 10.1016/j.ajog.2021.08.016 PMC8810751 34419453

[12] Benhalima, K. et al. Characteristics and pregnancy outcomes across gestational diabetes mellitus subtypes based on insulin resistance. *Diabetologia* **62**, 2118–2128 (2019). 31338546 10.1007/s00125-019-4961-7

[13] Kaul, P. et al. Association between maternal diabetes, being large for gestational age and breast-feeding on being overweight or obese in childhood. *Diabetologia* **62**, 249–258 (2019). 30421138 10.1007/s00125-018-4758-0

[14] Francis, E. C., Kechris, K., Jansson, T., Dabelea, D. & Perng, W. Novel metabolic subtypes in pregnant women and risk of early childhood obesity in offspring. *JAMA Netw. Open.* **6**, e237030 (2023). 37014638 10.1001/jamanetworkopen.2023.7030 PMC10074224

[15] Kahraman, S., Dirice, E., Jesus, D. F. D., Hu, J. & Kulkarni, R. N. Maternal insulin resistance and transient hyperglycemia impact the metabolic and endocrine phenotypes of offspring. *Am. J. Physiol. Endocrinol. Metabolism*. **307**, E906–E918 (2014). 10.1152/ajpendo.00210.2014 PMC4233258 25249504

[16] Jesus, D. F. D. et al. Parental metabolic syndrome epigenetically reprograms offspring hepatic lipid metabolism in mice. *J. Clin. Invest.* **130**, 2391–2407 (2020). 32250344 10.1172/JCI127502 PMC7190992

[17] Talton, O. O., Bates, K., Salazar, S. R., Ji, T. & Schulz, L. C. Lean maternal hyperglycemia alters offspring lipid metabolism and susceptibility to diet-induced obesity in mice†. *Biol. Reprod.* **100**, 1356–1369 (2019). 30698664 10.1093/biolre/ioz009

[18] Qiao, L. et al. Adiponectin deficiency impairs maternal metabolic adaptation to pregnancy in mice. *Diabetes* **66**, 1126–1135 (2017). 28073830 10.2337/db16-1096 PMC5399613

[19] Hufnagel, A., Dearden, L., Fernandez-Twinn, D. S. & Ozanne, S. E. Programming of cardiometabolic health: The role of maternal and fetal hyperinsulinaemia. *J. Endocrinol.* **253**, R47–R63 (2022). 35258482 10.1530/JOE-21-0332 PMC9066586

[20] Tol, A. J. C. et al. Hyperglycemia, pregnancy outcomes and maternal metabolic disease risk during pregnancy and lactation in a lean gestational diabetes mouse model. *J. Physiol.*10.1113/jp284061 (2023). 37010236 10.1113/JP284061

[21] Li, H. et al. A mouse model of gestation-specific transient hyperglycemia for translational studies. *J. Endocrinol.* **1**, 501–510 (2020). 10.1530/JOE-19-0516 31910155

[22] Hribar, K. et al. Postpartum development of metabolic dysfunction-associated steatotic liver disease in a lean mouse model of gestational diabetes mellitus. *Sci. Rep.* **14**, 14621 (2024). 38918525 10.1038/s41598-024-65239-2 PMC11199516

[23] Vieira-Lara, M. A. et al. Age and diet modulate the Insulin-Sensitizing effects of exercise: A Tracer-Based oral glucose tolerance test. *Diabetes* **72**, 872–883 (2023). 37204269 10.2337/db22-0746

[24] Dijk, T. H. et al. A novel approach to monitor glucose metabolism using stable isotopically labelled glucose in longitudinal studies in mice. *Lab. Anim.* **47**, 79–88 (2013). 23492513 10.1177/0023677212473714

[25] Dommerholt, M. B. et al. Short-term protein restriction at advanced age stimulates FGF21 signalling, energy expenditure and Browning of white adipose tissue. *Febs J.*10.1111/febs.15604 (2020). 33089625 10.1111/febs.15604 PMC8048886

[26] Lee, W. N., Byerley, L. O., Bergner, E. A. & Edmond, J. Mass isotopomer analysis: Theoretical and practical considerations. *Biol. Mass. Spectrom.* **20**, 451–458 (1991). 1768701 10.1002/bms.1200200804

[27] Vicini, P., Caumo, A. & Cobelli, C. The hot IVGTT two-compartment minimal model: Indexes of glucose effectiveness and insulin sensitivity. *Am. J. Physiol.* **273**, E1024–E1032 (1997). 9374690 10.1152/ajpendo.1997.273.5.E1024

[28] Matsuda, M. & DeFronzo, R. A. Insulin sensitivity indices obtained from oral glucose tolerance testing: Comparison with the euglycemic insulin clamp. *Diabetes Care*. **22**, 1462–1470 (1999). 10480510 10.2337/diacare.22.9.1462

[29] Bligh, E. G., Dyer, W. J., A rapid method of total lipid extraction and purification. *Can. J. Biochem. Physiol.* **37**, 911–917 (1959). 13671378 10.1139/o59-099

[30] Landon, M. B. et al. A multicenter, randomized trial of treatment for mild gestational diabetes. *N Engl. J. Med.* **361**, 1339–1348 (2009). 19797280 10.1056/NEJMoa0902430 PMC2804874

[31] Crowther, C. A. et al. Effect of treatment of gestational diabetes mellitus on pregnancy outcomes. *New. Engl. J. Med.* **352**, 2477–2486 (2005). 15951574 10.1056/NEJMoa042973

[32] Madsen, L. R. et al. Do variations in insulin sensitivity and insulin secretion in pregnancy predict differences in obstetric and neonatal outcomes? *Diabetologia* **64**, 304–312 (2021). 33156358 10.1007/s00125-020-05323-0

[33] Poissonnet, C. M., Burdi, A. R. & Bookstein, F. L. Growth and development of human adipose tissue during early gestation. *Early Hum. Dev.* **8**, 1–11 (1983). 6851910 10.1016/0378-3782(83)90028-2

[34] Kim, W., Park, S. K. & Kim, Y. L. Fetal abdominal overgrowth is already present at 20–24 gestational weeks prior to diagnosis of gestational diabetes mellitus. *Sci. Rep.* **11**, 23821 (2021). 34893662 10.1038/s41598-021-03145-7 PMC8664824

[35] Mihalovičová, L. et al. Severe gestational diabetes mellitus in lean dams is associated with low IL-1α levels and affects the growth of the juvenile mouse offspring. *Sci. Rep.* **13**, 1700 (2023). 36717684 10.1038/s41598-023-28903-7 PMC9886986

[36] Golic, M. et al. Diabetes mellitus in pregnancy leads to growth restriction and epigenetic modification of the Srebf2 gene in rat fetuses. *Hypertension* **71**, 911–920 (2018). 29610268 10.1161/HYPERTENSIONAHA.117.10782

[37] Barker, D. The developmental origins of chronic adult disease. *Acta Pædiatrica*. **93**, 26–33 (2004). 15702667 10.1111/j.1651-2227.2004.tb00236.x

[38] Lages, M., Barros, R., Moreira, P. & Guarino, M. P. Metabolic effects of an oral glucose tolerance test compared to the mixed meal tolerance tests: A narrative review. *Nutrients* **14**, 2032 (2022). 35631171 10.3390/nu14102032 PMC9147413

[39] Nuttall, F. Q., Gannon, M. C., Wald, J. L. & Ahmed, M. Plasma glucose and insulin profiles in normal subjects ingesting diets of varying carbohydrate, fat, and protein content. *J. Am. Coll. Nutr.* **4**, 437–450 (1985). 3900180 10.1080/07315724.1985.10720086

[40] Rijkelijkhuizen, J. M. et al. Effects of meal size and composition on incretin, α-cell, and β-cell responses. *Metabolism* **59**, 502–511 (2010). 19846181 10.1016/j.metabol.2009.07.039

[41] Horowitz, M. et al. Gastric and oesophageal emptying in patients with type 2 (non-insulin-dependent) diabetes mellitus. *Diabetologia* **32**, 151–159 (1989). 2753246 10.1007/BF00265086

[42] Prato, S. D. Loss of early insulin secretion leads to postprandial hyperglycaemia. *Diabetologia* **46**, M2–M8 (2003). 12652352 10.1007/s00125-002-0930-6

[43] Kowalski, G. M. & Bruce, C. R. The regulation of glucose metabolism: Implications and considerations for the assessment of glucose homeostasis in rodents. *Am. J. Physiol. -Endocrinol Metab.* **307**, E859–E871 (2014). 25205823 10.1152/ajpendo.00165.2014

[44] Abdul-Ghani, M. A., Matsuda, M., Balas, B. & DeFronzo, R. A. Muscle and liver insulin resistance indexes derived from the oral glucose tolerance test. *Diabetes Care*. **30**, 89–94 (2007). 17192339 10.2337/dc06-1519

[45] Jo, S. & Alejandro, E. Mechanistic insights into maternal-fetal crosstalk and islet beta-cell development. *J. Endocrinol.*10.1530/joe-23-0069 (2023). 37855321 10.1530/JOE-23-0069 PMC10692651

[46] Talbot, C. P. J. & Dolinsky, V. W. Sex differences in the developmental origins of cardiometabolic disease following exposure to maternal obesity and gestational diabetes. *Appl. Physiol. Nutr. Metab.* **44**, 687–695 (2019). 30500266 10.1139/apnm-2018-0667

[47] Dearden, L., Bouret, S. G. & Ozanne, S. E. Sex and gender differences in developmental programming of metabolism. *Mol. Metab.* **15**, 8–19 (2018). 29773464 10.1016/j.molmet.2018.04.007 PMC6066743

[48] Krishnaveni, G. V. et al. Intrauterine exposure to maternal diabetes is associated with higher adiposity and insulin resistance and clustering of cardiovascular risk markers in Indian children. *Diabetes Care*. **33**, 402–404 (2010). 19918007 10.2337/dc09-1393 PMC2809291

[49] Regnault, N., Gillman, M. W., Rifas-Shiman, S. L., Eggleston, E. & Oken, E. Sex-Specific associations of gestational glucose tolerance with childhood body composition. *Diabetes Care*. **36**, 3045–3053 (2013). 23877978 10.2337/dc13-0333 PMC3781569

