# Supplementary material for: Gestational hyperglycaemia impacts glucose control and insulin sensitivity in mouse offspring
Source: Sci Rep. 2025 Feb 28;15:7136. doi: 10.1038/s41598-025-91662-0 (PMC11871037; doi:10.1038/s41598-025-91662-0)
Supplement: Supplementary file 1 — Supplementary Material 1 [file 41598_2025_91662_MOESM1_ESM.docx]

**Supplementary Materials**

**Manuscript: Gestational hyperglycaemia impacts glucose control and insulin sensitivity in mouse offspring****.**

Hribar K, Fisher JC, Eichhorn D, Smit M, Kloosterhuis NJ , Bakker BM, Oosterveer MH, Kruit JK, van der Beek EM

**Tibia length measurements**

After termination, the left hind leg of the PN72 pups was removed and stored at -20C in PBS. The skin and other tissues surrounding the bones were removed after overnight soaking in lysis buffer (0.2mM Na_2_EDTA and 25mM NaOH). Tibia bone length was measured using a Mitutoyo IP67 ABSOLUTE Coolant-Proof Caliper.

**Plasma analysis**

Plasma lipid concentrations (triglycerides, cholesterol, and NEFA) were assessed using commercially available kits (Roche Diagnostics, Basel, Switzerland). Plasma leptin levels were analysed using a Mouse Leptin ELISA kit (Cat. #90030, Crystal Chem), according to the manufacturer’s protocol.

**Metabolic cage assessments**

Male and female offspring of CON and GDM dams at PN122 and PN310 of age were placed in metabolic monitoring systems (PromethION; Sable Systems International). Animals were singly housed with ad libitum access to chow diet and water and metabolic parameters were assessed for 3 days, consisting of three light and dark cycles of 12 h each. Animals were placed in cages in the middle of the light cycle, and data from the first light and dark cycles were excluded from the analysis to account for acclimation; data from the following 48 h were assessed. The cages assess food intake, energy expenditure, distance travelled, and respiratory quotient (RQ) using algorithms designed by the manufacturer (Sable Systems International). Food mass is weighed by sensors continuously to determine food intake. Energy expenditure is calculated according to the Weir equation: kcal per hour = 60*(0.003941*Vol O_2_ + 0.001106*Vol CO_2_), where VO_2_ and VCO_2_ are in mL/min. Distance travelled is the sum of all distances travelled within the beam brake system in meters, including fine movement (such as grooming and scratching) and direct locomotion. The respiratory quotient is calculated as Vol CO_2_/Vol O_2_.

**Mixed Meal Bolus**

The mixed meal bolus was prepared fresh using 4.57g of Nutridrink Powder Neutral (Nutricia Nederland, Zoetermeer; **Tbl. S2**) with the addition of 475 mg D-glucose and 50 mg stable isotope-labelled [U-¹³C₆]-glucose (tracer).

**Supplementary table 1. Nutritional value of Nutricia Nutridrink Neutral powder.** Ingredients include: Glucose syrup, SOY protein, vegetable oils (palm oil, sunflower oil, rapeseed oil), caseinate (from MILK), galacto-oligosaccharide (from MILK), tripotassium citrate, calcium carbonate, pectin, magnesium hydrogen phosphate, sodium chloride, flavoring (cream), fructo-oligosaccharide, emulsifier (SOYA lecithin), choline chloride, potassium chloride, L-ascorbic acid, sodium L-ascorbate, iron sulfate, zinc sulfate, nicotinamide, manganese sulfate, DL-a-tocopheryl acetate, calcium D-pantothenate, copper sulfate, retinyl acetate, D-biotin, pteroylmonoglutamic acid, cyanocobalamin, pyridoxine hydrochloride, thiamine hydrochloride, riboflavin, sodium fluoride, retinyl palmitate, DL-a-tocopherol, cholecalciferol, chromium chloride, sodium molybdate, potassium iodide, sodium selenite, phylloquinone. Nutritional value is shown as the absolute value per 100 g of the product.

| Energy | 1830 kJ / 435 kcal |
| --- | --- |
| Fats | 14,4 g |
| of which saturated | 3,4 g |
| Carbohydrates | 53,4 g |
| of which sugars | 8,6 g |
| Fibers | 2,6 g |
| Protein | 21,8 g |
| Vitamins |  |
| Vitamin A | 419 ug RE/ER |
| Vitamin D3 | 6,5 ug |
| Vitamin E | 5,2 mg a-TE/ET |
| Vitamin K | 23 ug |
| Thiamine | 0,58 mg |
| Riboflavin | 0,73 mg |
| Niacin | 4,2 mg NE/EN |
| Pantothenic acid | 2,3 mg |
| Vitamin B6 | 0,73 mg |
| Folic acid | 112 ug |
| Vitamin B12 | 1,2 ug |
| Biotin | 12 ug |
| Vitamin C | 44 mg |
| Minerals and trace elements |  |
| Na | 314 mg |
| K | 693 mg |
| Cl | 392 mg |
| Ca | 519 mg |
| P | 281 mg |
| Mg | 74 mg |
| Fe | 7 mg |
| Zn | 5,2 mg |
| Cu | 754 ug |
| Mn | 1,3 mg |
| F | 0,45 mg |
| Mo | 44 ug |
| Se | 26 ug |
| Cr | 20 ug |
| I | 56 ug |
| Other |  |
| Choline | 150 mg |
| Osmolarity | 330 mOsmol/l |
| Osmolality | 380 mOsmol/kg H2O |


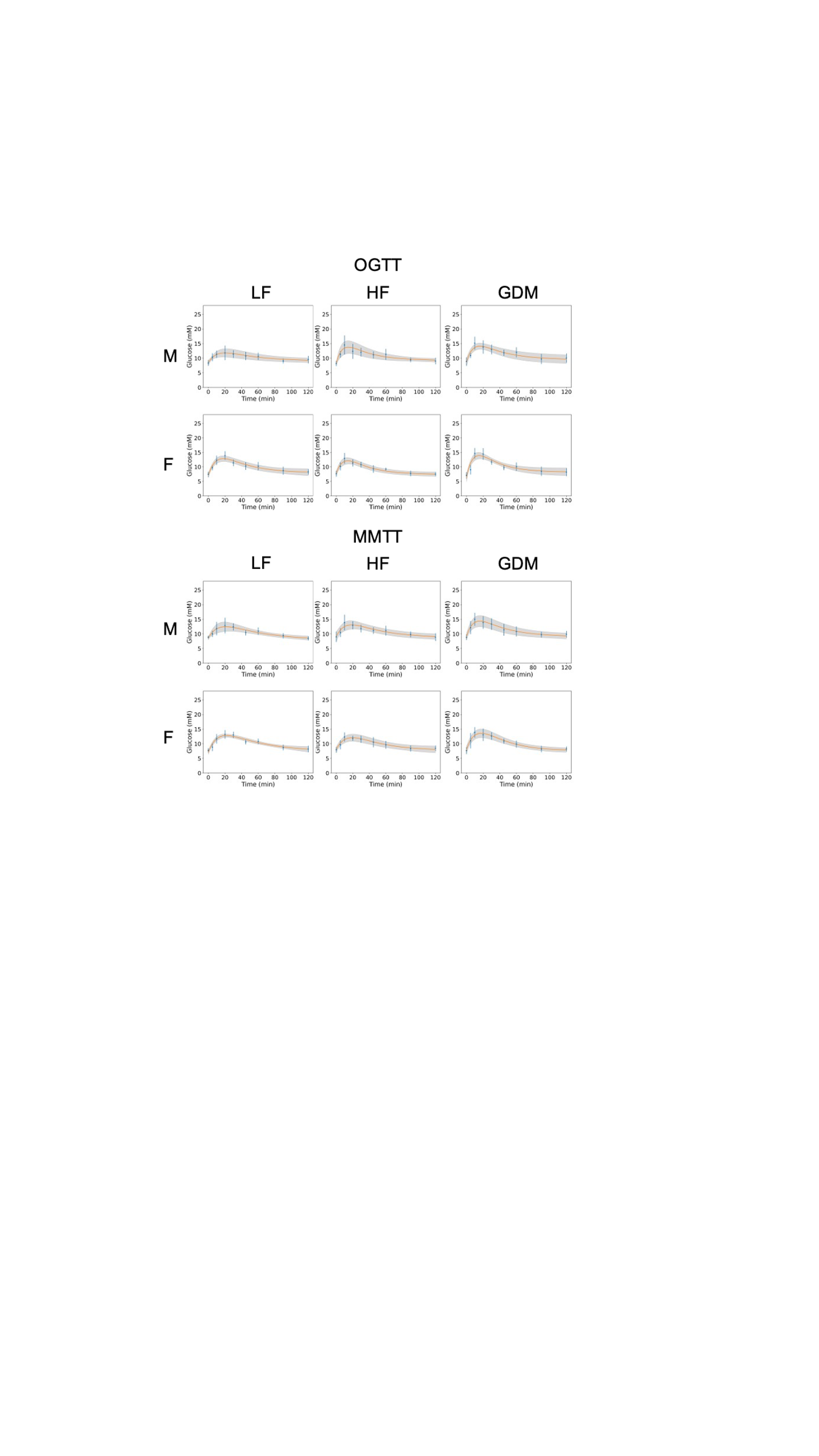


**Supplemental Figure 1. Curve fits for time courses of the [U-¹³C₆]-glucose tracer during OGTT/MMTT.** Each column represents a maternal exposure group, whereas each row represents a different sex. Sample size (offspring _dam_): male (LF:20_10_, HF:24_12_, GDM:26_13_) and female (LF:29_10_, HF:27 _12_, GDM:28_13_). Means ± SD for each time point are shown in blue. The average curve fit from all animals per experimental group (c2 in the model) is shown in orange, with the SD of the fitted curves represented by the shaded area.

**Supplemental table 2. Overview of absolute organ weights and plasma parameters at PN72 and 310.** Data are presented as mean ± SEM. Statistical analysis was performed using the One-way ANOVA followed by Tukey’s multiple comparison test. The overall p-values are presented in the final column. * GDM vs. LF, + GDM vs. HF, # HF vs. LF.

|  | LF | HF | GDM | p-value |
| --- | --- | --- | --- | --- |
| **PN72 male offspring** | **n=9** | **n=12** | **n=12** |  |
| Kidney (g) | 0.34 ± 0.01 | 0.34 ± 0.01 | 0.31 ± 0.01 | ns |
| pWAT (g) | 0.09 ± 0.02 | 0.15 ± 0.03 | 0.12 ± 0.02 | ns |
| gWAT (g) | 0.41 ± 0.04 | 0.53 ± 0.07 | 0.41 ± 0.04 | ns |
| sWAT (g) | 0.25 ± 0.02 | 0.38 ± 0.04 | 0.28 ± 0.03 | 0.0160 |
| BAT (g) | 0.13 ± 0.01 | 0.17 ± 0.02 | 0.12 ± 0.01 | ns |
| Liver (g) | 1.13 ± 0.06 | 1.25 ± 0.05 | 1.11 ± 0.04 | ns |
| Brain (g) | 0.44 ± 0.01 | 0.44 ± 0.01 | 0.43 ± 0.01 | ns |
| Heart (g) | 0.11 ± 0.01 | 0.11 ± 0.00 | 0.10 ± 0.00 | ns |
| Skeletal muscle (g) | 0.26 ± 0.04 | 0.31 ± 0.02 | 0.27 ± 0.02 | ns |
| Tibia (mm) | 20.25 ± 1.02 | 20.36 ± 0.59 | 21.32 ± 0.72 | ns |
| Femour (mm) | 13.12 ± 0.48 | 13.70 ± 0.29 | 14.17 ± 0.33 | ns |
| Non-Esterified FA (mmol/L) | 0.62 ± 0.06 | 0.98 ± 0.10 | 0.78 ± 0.05 | ns |
| Cholesterol (mmol/L) | 2.47 ± 0.13 | 2.69 ± 0.19 | 2.67 ± 0.24 | ns |
| Triglycerides (mmol/L) | 0.25 ± 0.04 | 0.36 ± 0.05 | 0.43 ± 0.09 | ns |
| Leptin (ng/mL) | 3.50 ± 0.88 | 15.54 ± 7.72 | 2.70 ± 0.64 | ns |
| **PN72 Female offspring** | **n=9** | **n=7** | **n=8** |  |
| Kidney (g) | 0.26 ± 0.01 | 0.26 ± 0.02 | 0.26 ± 0.01 | ns |
| pWAT (g) | 0.07 ± 0.01 | 0.07 ± 0.02 | 0.09 ± 0.01 | ns |
| gWAT (g) | 0.20 ± 0.02 | 0.15 ± 0.02 | 0.16 ± 0.02 | ns |
| sWAT (g) | 0.18 ± 0.01 | 0.20 ± 0.02 | 0.20 ± 0.01 | ns |
| BAT (g) | 0.10 ± 0.01 | 0.09 ± 0.02 | 0.11 ± 0.01 | ns |
| Liver (g) | 0.93 ± 0.03 | 0.84 ± 0.04 | 0.80 ± 0.06 | ns |
| Brain (g) | 0.43 ± 0.00 | 0.43 ± 0.01 | 0.43 ± 0.01 | ns |
| Heart (g) | 0.09 ± 0.00 | 0.09 ± 0.01 | 0.09 ± 0.01 | ns |
| Skeletal muscle (g) | 0.18 ± 0.03 | 0.25 ± 0.00 | 0.23 ± 0.01 | ns |
| Tibia (mm) | 21.11 ± 0.62 | 20.97 ± 0.66 | 20.35 ± 0.46 | ns |
| Femour (mm) | 14.62 ± 0.27 | 13.86 ± 0.62 | 13.66 ± 0.22 | ns |
| Non-Esterified FA (mmol/L) | 0.64 ± 0.05 | 0.58 ± 0.11 | 0.64 ± 0.06 | ns |
| Cholesterol (mmol/L) | 2.07 ± 0.12 | 2.09 ± 16 | 1.62 ± 0.07 | ns |
| Triglycerides (mmol/L) | 0.34 ± 0.12 | 0.28 ± 0.06 | 0.17 ± 0.04 | ns |
| Leptin (ng/mL) | 5.25 ± 3.28 | 9.10 ± 4.01 | 5.36 ± 3.69 | ns |
| **PN310 male offspring** | **n=9** | **n=11** | **n=13** |  |
| Liver (g) | 1.81 ± 0.04 | 1.84 ± 0.11 | 1.73 ± 0.07 | ns |
| Pancreas (g) | 0.26 ± 0.01 | 0.26 ± 0.01 | 0.27 ± 0.01 | ns |
| sWAT (g) | 1.52 ± 0.08 | 1.42 ± 0.12 | 1.43 ± 0.14 | ns |
| pWAT (g) | 0.91 ± 0.06 | 0.85 ± 0.08 | 0.87 ± 0.04 | ns |
| gWAT (g) | 1.88 ± 0.11 | 1.87 ± 0.16 | 1.78 ± 0.14 | ns |
| BAT (g) | 0.33 ± 0.02 | 0.34 ± 0.04 | 0.31 ± 0.02 | ns |
| Brain (g) | 0.45 ± 0.00 | 0.42 ± 0.04 | 0.44 ± 0.01 | ns |
| Heart (g) | 0.17 ± 0.01 | 0.16 ± 0.00 | 0.15 ± 0.00 | ns |
| Skeletal muscle (g) | 0.18 ± 0.00 | 0.18 ± 0.00 | 0.18 ± 0.00 | ns |
| Non-Esterified FA (mmol/L) | 0.76 ± 0.05 | 0.68 ± 0.04 | 0.72 ± 0.03 | ns |
| Cholesterol (mmol/L) | 2.49 ± 0.05 | 2.47 ± 0.07 | 2.34 ± 0.14 | ns |
| Triglycerides (mmol/L) | 1.11 ± 0.29 | 0.93 ± 0.48 | 0.44 ± 0.11 | ns |
| Leptin (ng/mL) | 5.30 ± 1.11 | 5.28 ± 1.18 | 5.47 ± 1.08 | ns |
| **PN310 female offspring** | **n=9** | **n=11** | **n=12** |  |
| Liver (g) | 1.48 ± 0.08 | 1.49 ± 0.04 | 1.39 ± 0.11 | ns |
| Pancreas (g) | 0.28 ± 0.01 | 0.27 ± 0.01 | 0.30 ± 0.01 | ns |
| sWAT (g) | 1.33 ± 0.20 | 1.06 ± 0.10 | 1.16 ± 0.18 | ns |
| pWAT (g) | 1.15 ± 0.20 | 0.87 ± 0.10 | 0.87 ± 0.15 | ns |
| gWAT (g) | 1.77 ± 0.33 | 1.37 ± 0.13 | 1.23 ± 0.24 | ns |
| BAT (g) | 0.21 ± 0.03 | 0.19 ± 0.02 | 0.21 ± 0.03 | ns |
| Brain (g) | 0.45 ± 0.01 | 0.45 ± 0.01 | 0.45 ± 0.01 | ns |
| Heart (g) | 0.15 ± 0.00 | 0.14 ± 0.00 | 0.15 ± 0.00 | ns |
| Skeletal muscle (g) | 0.15 ± 0.01 | 0.16 ± 0.01 | 0.16 ± 0.01 | ns |
| Non-Esterified FA (mmol/L) | 0.82 ± 0.04 | 0.86 ± 0.10 | 0.66 ± 0.04 | ns |
| Cholesterol (mmol/L) | 2.02 ± 0.04 | 2.44 ± 0.25 | 1.96 ± 0.13 | ns |
| Triglycerides (mmol/L) | 0.70 ± 0.22 | 1.10 ± 0.48 | 0.42 ± 0.10 | ns |
| Leptin (ng/mL) | 5.52 ± 1.15 | 4.36 ± 0.34 | 6.11 ± 0.70 | ns |

**
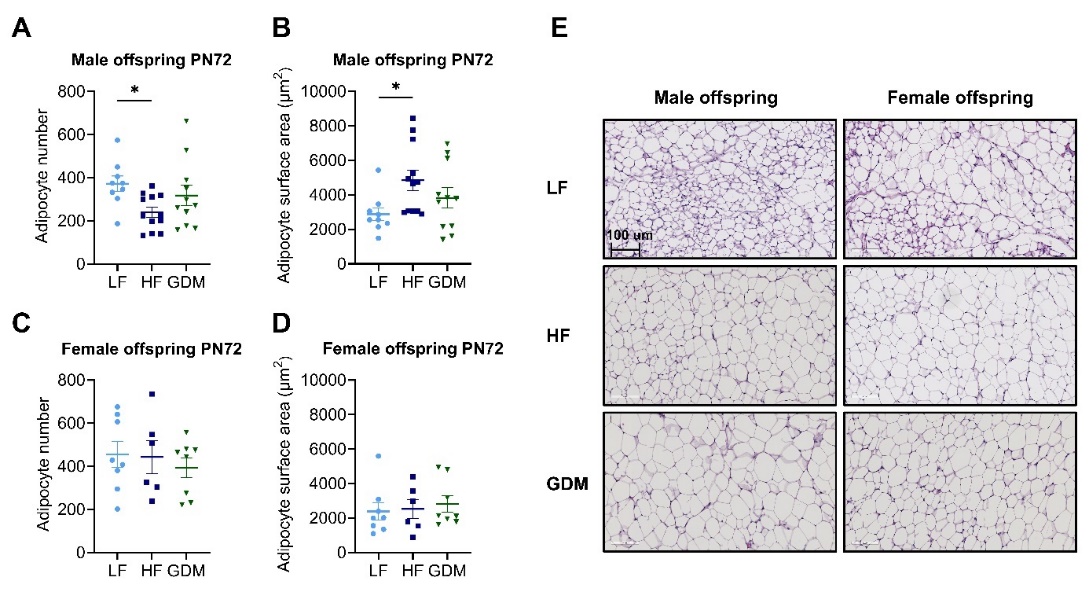
**

**Supplemental Figure 2. sWAT adipocyte number and surface area in male and female offspring at PN72.** sWAT Adipocyte number in sWAT of male (**A**) and female offspring (**C**). Adipocyte surface area (μm2) in sWAT of male (**B**) and female offspring (**C**). Representative sWAT H&E stained sections (**E**). Sample size (offspring _dam_): male (LF:20_10_, HF:24_12_, GDM:26_13_) and female (LF:29_10_, HF:27 _12_, GDM:28_13_). Data are presented as the mean ± SEM. **A-D:** One-way ANOVA followed by Tukey’s multiple comparison test. *p<0.05, **p<0.01, ***p<0.001, ****p<0.0001.

**
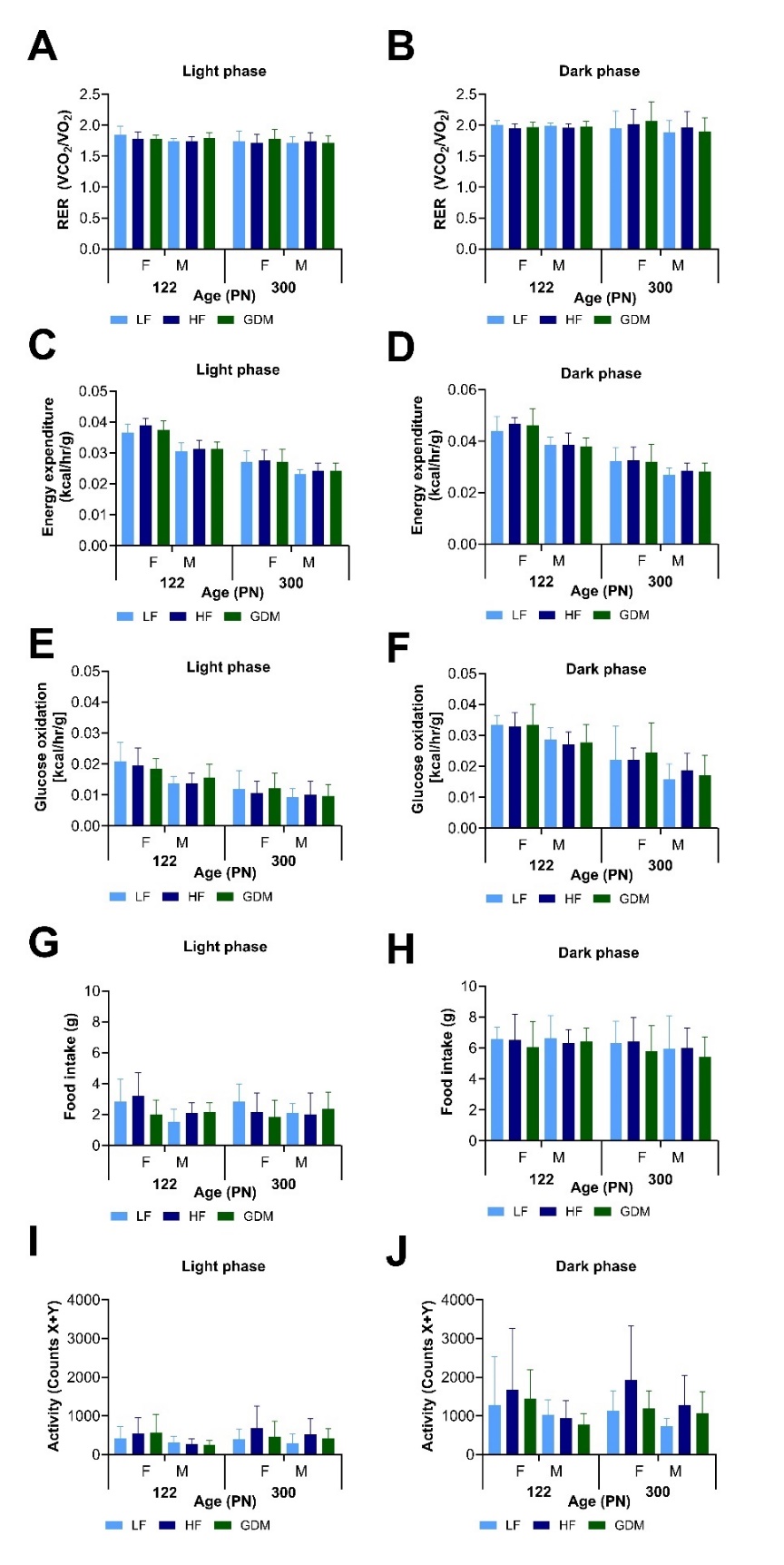
**

**Supplemental Figure 3. Female and male offspring respiratory exchange ratio, energy expenditure, glucose oxidation, food intake, and activity at PN122 and 300.** Respiratory exchange ratio (RER) during light (**A**) and dark (**B**) phases. Energy expenditure during the light (**C**) and dark (**D**) phases. Glucose oxidation during light (**E**) and dark (**F**) phases. Food intake during light (**G**) and dark (**H**) phases. Activity during light (**I**) and dark (**J**) phases. Sample size: male (LF:10 _10_, HF:11_12_, GDM:13_13_), female (LF:9_10_, HF:11 _12_, GDM:12_13_). Data are presented as the mean ± SD. **A-C:** One-way ANOVA followed by Tukey’s multiple comparison test. * GDM vs. LF, + GDM vs. HF, # HF vs. LF. *p<0.05, **p<0.01, ***p<0.001, ****p<0.0001.


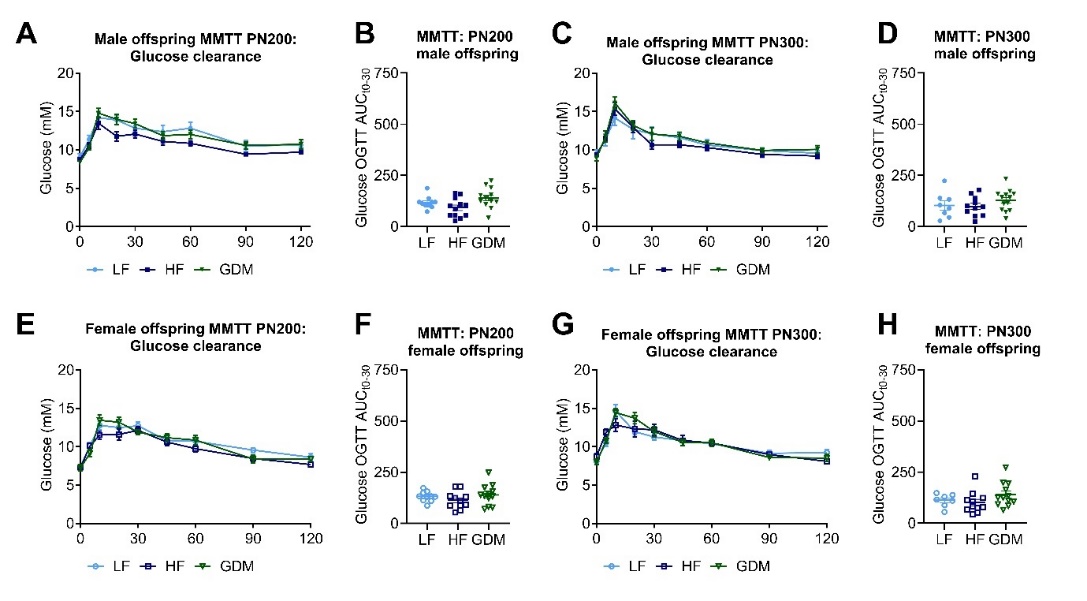


**Supplemental figure 4. Glucose clearance in male and female offspring during MMTT at PN200 and 300.** Absolute glucose values during MMTT PN200/300 in male (**A/C**) and female (**E/G**) offspring. AUC for glucose clearance induced by MMTT PN200/300 (t0-30) in male (**B/D**) and female (**F/H**) offspring. Sample size (offspring _dam_): male (LF:10 _10_, HF:11 _12_, GDM:13 _13_), female (LF:9 _10_, HF:11 _12_, GDM:12 _13_). Data are presented as the mean ± SEM, with each value representing the average of the measurement of the individual offspring from one litter. **A, C, E, G:** Mixed effects 2-way ANOVA followed by Tukey’s multiple comparison test were used. **B, D, F, H:** Ordinary 1-way ANOVA analysis followed by Tukey’s multiple comparison test was used. * GDM vs. LF, + GDM vs. HF, # HF vs. LF. *p<0.05, **p<0.01, ***p<0.001, ****p<0.0001.

**Supplemental table 3. PN95/100, 195/200 and 219/300 OGTT and MMTT glucose, OGTT insulin.** Data are presented as the mean ± SEM. Mixed effects 2-way ANOVA followed by Tukey’s multiple comparison test were used. B, D, F, H: Ordinary 1-way ANOVA analysis followed by Tukey’s multiple comparison test was used. LF, low-fat diet; HF, high-fat diet; GDM, gestational diabetes mellitus.

|  | **LF** | **HF** | **GDM** | **LF vs. HF** | **LF vs. GDM** | **HF vs. GDM** |
| --- | --- | --- | --- | --- | --- | --- |
| **PN95/100** |  |  |  |  |  |  |
| **Males** |  |  |  |  |  |  |
| OGTT Glucose clearance (mM) |  |  |  |  |  |  |
| t0 | 8.41 ± 0.36 | 8.46 ± 0.28 | 8.7 ± 0.41 | ns | ns | ns |
| t5 | 10.34 ± 0.51 | 11.25 ± 0.37 | 11.02 ± 0.25 | ns | ns | ns |
| t10 | 11.31 ± 0.45 | 14.99 ± 1.1 | 15.27 ± 0.66 | 0.0116 | 0.0002 | ns |
| t20 | 12 ± 0.95 | 12.95 ± 0.87 | 13.88 ± 0.69 | ns | ns | ns |
| t30 | 11.48 ± 0.48 | 12.37 ± 0.51 | 13.19 ± 0.52 | ns | ns | ns |
| t45 | 10.77 ± 0.53 | 11.54 ± 0.4 | 11.88 ± 0.31 | ns | ns | ns |
| t60 | 10.58 ± 0.47 | 11.7 ± 0.61 | 12.08 ± 0.51 | ns | ns | ns |
| t90 | 9.06 ± 0.3 | 9.65 ± 0.26 | 9.58 ± 0.51 | ns | ns | ns |
| t120 | 9.57 ± 0.53 | 9.26 ± 0.37 | 10.01 ± 0.45 | ns | ns | ns |
| OGTT Insulin release (mU/L) |  |  |  |  |  |  |
| t0 | 11.15 ± 1.89 | 11.27 ± 1.83 | 10.56 ± 1.55 | ns | ns | ns |
| t5 | 14.84 ± 1.44 | 17.48 ± 2.32 | 15.75 ± 1.56 | ns | ns | ns |
| t10 | 21.05 ± 5.1 | 20.21 ± 3.1 | 18.97 ± 2.1 | ns | ns | ns |
| t30 | 15.17 ± 2.14 | 12.98 ± 1.17 | 14.83 ± 2.67 | ns | ns | ns |
| t60 | 13.56 ± 1.08 | 14.16 ± 1.22 | 13.52 ± 1.65 | ns | ns | ns |
| t120 | 14.27 ± 1.96 | 13.3 ± 1.48 | 14.18 ± 2.95 | ns | ns | ns |
| MMTT Glucose clearance (mM) |  |  |  |  |  |  |
| t0 | 8.83 ± 0.2 | 9.13 ± 0.58 | 8.98 ± 0.29 | ns | ns | ns |
| t5 | 10 ± 0.4 | 10.48 ± 0.53 | 11.97 ± 0.67 | ns | 0.0495 | ns |
| t10 | 11.61 ± 0.83 | 14.28 ± 0.92 | 14.87 ± 0.67 | ns | 0.0191 | ns |
| t20 | 13.03 ± 0.97 | 12.95 ± 0.48 | 13.93 ± 0.66 | ns | ns | ns |
| t30 | 12.38 ± 0.46 | 11.63 ± 0.43 | 13.27 ± 0.59 | ns | ns | ns |
| t45 | 10.34 ± 0.34 | 10.93 ± 0.38 | 11.48 ± 0.61 | ns | ns | ns |
| t60 | 10.89 ± 0.47 | 11.2 ± 0.58 | 10.93 ± 0.51 | ns | ns | ns |
| t90 | 9.27 ± 0.32 | 9.83 ± 0.35 | 9.73 ± 0.3 | ns | ns | ns |
| t120 | 8.37 ± 0.23 | 9.15 ± 0.46 | 9.77 ± 0.34 | ns | 0.0071 | ns |
| MMTT Insulin release (mU/L) |  |  |  |  |  |  |
| t0 | 14.24 ± 2.85 | 16.53 ± 2.6 | 11.6 ± 1.53 | ns | ns | ns |
| t5 | 17.09 ± 2.4 | 15.81 ± 2.4 | 12.38 ± 1.59 | ns | ns | ns |
| t10 | 16.78 ± 2.39 | 16.09 ± 4.48 | 11.34 ± 1.46 | ns | ns | ns |
| t30 | 20.59 ± 3.43 | 11.61 ± 1.38 | 11.11 ± 1.4 | ns | 0.0295 |  |
| t60 | 18.65 ± 2.74 | 15.94 ± 2.17 | 12.05 ± 2.23 | ns | ns | ns |
| t120 | 17.32 ± 3.18 | 12.67 ± 1.53 | 14.58 ± 2.35 | ns | ns | ns |
| **Females** |  |  |  |  |  |  |
| OGTT Glucose clearance (mM) |  |  |  |  |  |  |
| t0 | 7.54 ± 0.31 | 7.65 ± 0.33 | 7.08 ± 0.33 | ns | ns | ns |
| t5 | 9.9 ± 0.3 | 10.25 ± 0.45 | 9.06 ± 0.51 | ns | ns | ns |
| t10 | 12.76 ± 0.6 | 13.13 ± 0.67 | 14.93 ± 0.65 | ns | 0.031 | ns |
| t20 | 14.38 ± 0.6 | 11.58 ± 0.41 | 14.76 ± 0.63 | 0.0059 | ns | 0.0014 |
| t30 | 11.59 ± 0.42 | 11 ± 0.32 | 11.94 ± 0.3 | ns | ns | ns |
| t45 | 10.53 ± 0.51 | 9.43 ± 0.42 | 9.92 ± 0.27 | ns | ns | ns |
| t60 | 10.61 ± 0.48 | 9.35 ± 0.17 | 10.2 ± 0.45 | ns | ns | ns |
| t90 | 9.12 ± 0.4 | 7.81 ± 0.32 | 8.55 ± 0.52 | ns | ns | ns |
| t120 | 8.56 ± 0.23 | 7.55 ± 0.33 | 8.26 ± 0.44 | ns | ns | ns |
| OGTT Insulin release (mU/L) |  |  |  |  |  |  |
| t0 | 14.28 ± 1.75 | 8.68 ± 0.88 | 7.38 ± 1.02 | 0.0178 | 0.0061 | ns |
| t5 | 16.47 ± 2.95 | 12.01 ± 1.97 | 12.42 ± 2.27 | ns | ns | ns |
| t10 | 15.91 ± 2.12 | 13.45 ± 1.3 | 11.77 ± 1.26 | ns | ns | ns |
| t30 | 16.82 ± 2.91 | 19.38 ± 10.13 | 8.8 ± 1.15 | ns | ns | ns |
| t60 | 13.22 ± 1.49 | 16.77 ± 7.5 | 11.44 ± 2.08 | ns | ns | ns |
| t120 | 13.91 ± 1.31 | 9.53 ± 1.34 | 16.01 ± 4.22 | ns | ns | ns |
| MMTT Glucose clearance (mM) |  |  |  |  |  |  |
| t0 | 7.78 ± 0.29 | 7.93 ± 0.32 | 7.68 ± 0.37 | ns | ns | ns |
| t5 | 9.01 ± 0.48 | 9.64 ± 0.51 | 11.06 ± 0.79 | ns | ns | ns |
| t10 | 11.51 ± 0.57 | 12.33 ± 0.53 | 13.89 ± 0.54 | ns | 0.0249 | ns |
| t20 | 13.03 ± 0.52 | 11.88 ± 0.28 | 13.06 ± 0.64 | ns | ns | ns |
| t30 | 13.12 ± 0.39 | 11.57 ± 0.41 | 12.63 ± 0.39 | 0.0372 | ns | ns |
| t45 | 10.61 ± 0.31 | 10.54 ± 0.58 | 10.91 ± 0.26 | ns | ns | ns |
| t60 | 10.82 ± 0.35 | 9.62 ± 0.45 | 9.88 ± 0.37 | ns | ns | ns |
| t90 | 8.68 ± 0.37 | 8.36 ± 0.35 | 8.22 ± 0.33 | ns | ns | ns |
| t120 | 8.1 ± 0.39 | 8.46 ± 0.31 | 8.22 ± 0.24 | ns | ns | ns |
| MMTT Insulin release (mU/L) |  |  |  |  |  |  |
| t0 | 12.64 ± 1.33 | 10.45 ± 2.7 | 8.88 ± 2.2 | ns | ns | ns |
| t5 | 13.4 ± 1.84 | 9.87 ± 2.6 | 9.39 ± 1.84 | ns | ns | ns |
| t10 | 14.56 ± 2.7 | 9.42 ± 1.93 | 7.68 ± 0.87 | 0.044 | 0.0044 | ns |
| t30 | 27.26 ± 12.38 | 8.55 ± 1.56 | 8.01 ± 0.83 | 0.0309 | 0.0127 | ns |
| t60 | 13.27 ± 1.42 | 8.47 ± 1.78 | 8.22 ± 1.19 | 0.0432 | 0.0255 | ns |
| t120 | 13.41 ± 1.24 | 8.76 ± 1.98 | 7.51 ± 1.06 | ns | 0.0148 | ns |
| **PN195/200** |  |  |  |  |  |  |
| **Males** |  |  |  |  |  |  |
| OGTT Glucose clearance (mM) |  |  |  |  |  |  |
| t0 | 8.53 ± 0.5 | 8.44 ± 0.33 | 8.53 ± 0.21 | ns | ns | ns |
| t5 | 11.72 ± 0.61 | 11.53 ± 0.37 | 11.55 ± 0.38 | ns | ns | ns |
| t10 | 16.18 ± 0.62 | 17.25 ± 0.79 | 17.92 ± 0.7 | ns | ns | ns |
| t20 | 13.28 ± 0.62 | 14.17 ± 0.37 | 14.14 ± 0.9 | ns | ns | ns |
| t30 | 13.13 ± 0.69 | 12.08 ± 0.32 | 13.24 ± 0.6 | ns | ns | ns |
| t45 | 13.26 ± 0.72 | 11.9 ± 0.27 | 13.75 ± 0.57 | ns | ns | 0.0185 |
| t60 | 13.21 ± 0.69 | 12.45 ± 0.6 | 13.56 ± 0.52 | ns | ns | ns |
| t90 | 11.91 ± 0.49 | 10.96 ± 0.44 | 11.39 ± 0.5 | ns | ns | ns |
| t120 | 10.51 ± 0.79 | 10.05 ± 0.3 | 10.6 ± 0.63 | ns | ns | ns |
| OGTT Insulin release (mU/L) |  |  |  |  |  |  |
| t0 | 10.07 ± 1.41 | 15.09 ± 3.05 | 14.12 ± 2.79 | ns | ns | ns |
| t5 | 20.97 ± 4.38 | 28.15 ± 8.58 | 22.48 ± 4.83 | ns | ns | ns |
| t10 | 20.3 ± 2.19 | 26.54 ± 5.5 | 30.94 ± 10.25 | ns | ns | ns |
| t30 | 20.78 ± 4.52 | 15.01 ± 2.28 | 14.97 ± 3.26 | ns | ns | ns |
| t60 | 20.45 ± 4.85 | 16.82 ± 3.29 | 13.85 ± 2.85 | ns | ns | ns |
| t120 | 20.1 ± 3.49 | 21.12 ± 4.64 | 14.43 ± 2.84 | ns | ns | ns |
| MMTT Glucose clearance (mM) |  |  |  |  |  |  |
| t0 | 9.14 ± 0.5 | 8.71 ± 0.28 | 8.35 ± 0.29 | ns | ns | ns |
| t5 | 11.42 ± 0.62 | 10.68 ± 0.34 | 10.27 ± 0.31 | ns | ns | ns |
| t10 | 14.37 ± 0.79 | 13.74 ± 0.82 | 14.78 ± 0.62 | ns | ns | ns |
| t20 | 14.01 ± 0.7 | 11.83 ± 0.66 | 13.98 ± 0.67 | ns | ns | ns |
| t30 | 12.74 ± 0.4 | 12.31 ± 0.49 | 13.43 ± 0.59 | ns | ns | ns |
| t45 | 12.64 ± 0.84 | 11.17 ± 0.52 | 11.86 ± 0.54 | ns | ns | ns |
| t60 | 12.91 ± 0.85 | 10.84 ± 0.36 | 12.03 ± 0.53 | ns | ns | ns |
| t90 | 10.26 ± 0.79 | 9.36 ± 0.23 | 10.59 ± 0.44 | ns | ns | ns |
| t120 | 10.53 ± 0.35 | 9.7 ± 0.22 | 10.72 ± 0.6 | ns | ns | ns |
| **Females** |  |  |  |  |  |  |
| OGTT Glucose clearance (mM) |  |  |  |  |  |  |
| t0 | 6.93 ± 0.37 | 7.12 ± 0.22 | 7.13 ± 0.32 | ns | ns | ns |
| t5 | 11.16 ± 1.02 | 11.35 ± 0.58 | 10.17 ± 0.44 | ns | ns | ns |
| t10 | 15.11 ± 0.72 | 14.87 ± 0.62 | 15.03 ± 0.42 | ns | ns | ns |
| t20 | 13.19 ± 0.5 | 13.25 ± 0.68 | 13.1 ± 0.49 | ns | ns | ns |
| t30 | 11.83 ± 0.46 | 11.83 ± 0.53 | 12.53 ± 0.78 | ns | ns | ns |
| t45 | 11.01 ± 0.71 | 9.83 ± 0.29 | 10.21 ± 0.55 | ns | ns | ns |
| t60 | 10.31 ± 0.45 | 10.37 ± 0.18 | 10.5 ± 0.76 | ns | ns | ns |
| t90 | 8.28 ± 0.4 | 8.29 ± 0.23 | 8.34 ± 0.5 | ns | ns | ns |
| t120 | 8.48 ± 0.28 | 7.93 ± 0.14 | 7.96 ± 0.36 | ns | ns | ns |
| OGTT Insulin release (mU/L) |  |  |  |  |  |  |
| t0 | 10.22 ± 1.94 | 8.96 ± 1.08 | 9.49 ± 1.26 | ns | ns | ns |
| t5 | 15.21 ± 3.99 | 12.31 ± 2.46 | 13.98 ± 2.17 | ns | ns | ns |
| t10 | 20.69 ± 3.89 | 12.88 ± 1.7 | 13.36 ± 2.51 | ns | ns | ns |
| t30 | 13.09 ± 1.7 | 13.22 ± 2.7 | 22.14 ± 7.83 | ns | ns | ns |
| t60 | 14.59 ± 2.95 | 9.79 ± 1.5 | 16.25 ± 3.99 | ns | ns | ns |
| t120 | 11.83 ± 1.76 | 9.56 ± 0.81 | 12.49 ± 3.13 | ns | ns | ns |
| MMTT Glucose clearance (mM) |  |  |  |  |  |  |
| t0 | 7.32 ± 0.21 | 7.31 ± 0.24 | 7.24 ± 0.42 | ns | ns | ns |
| t5 | 10.14 ± 0.45 | 10.13 ± 0.35 | 9.18 ± 0.43 | ns | ns | ns |
| t10 | 12.44 ± 0.48 | 11.52 ± 0.52 | 13.45 ± 0.69 | ns | ns | ns |
| t20 | 12.36 ± 0.88 | 11.61 ± 0.74 | 13.19 ± 0.65 | ns | ns | ns |
| t30 | 12.97 ± 0.51 | 12.13 ± 0.5 | 11.88 ± 0.34 | ns | ns | ns |
| t45 | 10.62 ± 0.43 | 10.6 ± 0.43 | 11.17 ± 0.51 | ns | ns | ns |
| t60 | 10.68 ± 0.58 | 9.75 ± 0.4 | 10.86 ± 0.62 | ns | ns | ns |
| t90 | 9.71 ± 0.32 | 8.46 ± 0.37 | 8.39 ± 0.48 | ns | ns | ns |
| t120 | 8.27 ± 0.32 | 7.67 ± 0.28 | 8.38 ± 0.34 | ns | ns | ns |
| **PN295/300** |  |  |  |  |  |  |
| **Males** |  |  |  |  |  |  |
| OGTT Glucose clearance (mM) |  |  |  |  |  |  |
| t0 | 8.52 ± 0.53 | 8.75 ± 0.2 | 8.72 ± 0.4 | ns | ns | ns |
| t5 | 11.31 ± 0.86 | 12.28 ± 0.5 | 12.04 ± 0.57 | ns | ns | ns |
| t10 | 16.14 ± 0.75 | 17.59 ± 0.8 | 19.1 ± 0.82 | ns | 0.0072 | ns |
| t20 | 16.01 ± 1.03 | 14.68 ± 0.73 | 17.41 ± 1.13 | ns | ns | ns |
| t30 | 12.58 ± 0.43 | 11.92 ± 0.38 | 14.22 ± 0.75 | ns | ns | 0.0228 |
| t45 | 13.86 ± 0.73 | 13.05 ± 0.41 | 13.99 ± 0.6 | ns | ns | ns |
| t60 | 13.07 ± 0.67 | 12.48 ± 0.4 | 13.59 ± 0.72 | ns | ns | ns |
| t90 | 11.94 ± 0.62 | 11.35 ± 0.31 | 12.18 ± 0.66 | ns | ns | ns |
| t120 | 11.53 ± 0.43 | 11.67 ± 0.54 | 12.05 ± 0.57 | ns | ns | ns |
| OGTT Insulin release (mU/L) |  |  |  |  |  |  |
| t0 | 17.52 ± 1.78 | 11.54 ± 1.35 | 15.14 ± 3.73 | ns | ns | ns |
| t5 | 65.83 ± 12.6 | 34.2 ± 4.78 | 38.03 ± 10.08 | ns | ns | ns |
| t10 | 36.55 ± 4.68 | 48.42 ± 10.45 | 48.45 ± 17.96 | ns | ns | ns |
| t30 | 31.69 ± 4.32 | 21.02 ± 4.3 | 18.85 ± 5.69 | ns | ns | ns |
| t60 | 33.9 ± 6.71 | 20.38 ± 4.7 | 19.68 ± 3.94 | ns | ns | ns |
| t120 | 34.98 ± 4.41 | 26.02 ± 3.13 | 28.17 ± 8.66 | ns | ns | ns |
| MMTT Glucose clearance (mM) |  |  |  |  |  |  |
| t0 | 9.58 ± 0.51 | 9.37 ± 0.21 | 8.9 ± 0.33 | ns | ns | ns |
| t5 | 11.29 ± 0.88 | 11.4 ± 0.37 | 11.76 ± 0.73 | ns | ns | ns |
| t10 | 14.31 ± 1.1 | 15.32 ± 0.85 | 16.12 ± 0.82 | ns | ns | ns |
| t20 | 12.4 ± 1.3 | 12.93 ± 0.68 | 13.19 ± 0.62 | ns | ns | ns |
| t30 | 11.64 ± 0.89 | 10.68 ± 0.6 | 12.08 ± 0.79 | ns | ns | ns |
| t45 | 11.18 ± 0.39 | 10.67 ± 0.34 | 11.78 ± 0.53 | ns | ns | ns |
| t60 | 10.54 ± 0.86 | 10.31 ± 0.36 | 10.93 ± 0.25 | ns | ns | ns |
| t90 | 9.88 ± 0.4 | 9.43 ± 0.38 | 9.94 ± 0.31 | ns | ns | ns |
| t120 | 9.36 ± 0.33 | 9.2 ± 0.33 | 10.08 ± 0.51 | ns | ns | ns |
| **Females** |  |  |  |  |  |  |
| OGTT Glucose clearance (mM) |  |  |  |  |  |  |
| t0 | 7.54 ± 0.35 | 7.7 ± 0.34 | 7.49 ± 0.32 | ns | ns | ns |
| t5 | 11.4 ± 0.83 | 12.15 ± 0.72 | 12.05 ± 0.76 | ns | ns | ns |
| t10 | 16.44 ± 1.14 | 16.17 ± 0.69 | 16.67 ± 0.78 | ns | ns | ns |
| t20 | 13.23 ± 0.74 | 12.99 ± 0.5 | 13.07 ± 0.49 | ns | ns | ns |
| t30 | 12.43 ± 0.47 | 12.18 ± 0.53 | 12.25 ± 0.53 | ns | ns | ns |
| t45 | 11.09 ± 0.51 | 11.29 ± 0.56 | 11.45 ± 0.51 | ns | ns | ns |
| t60 | 10.93 ± 0.56 | 10.62 ± 0.31 | 10.71 ± 0.29 | ns | ns | ns |
| t90 | 9.34 ± 0.49 | 9.64 ± 0.42 | 9.4 ± 0.5 | ns | ns | ns |
| t120 | 8.81 ± 0.29 | 8.88 ± 0.32 | 8.95 ± 0.32 | ns | ns | ns |
| OGTT Insulin release (mU/L) |  |  |  |  |  |  |
| t0 | 11.69 ± 3.01 | 14.25 ± 2.63 | 14.22 ± 2.63 | ns | ns | ns |
| t5 | 20.77 ± 4.76 | 29.07 ± 8.33 | 27.89 ± 8.47 | ns | ns | ns |
| t10 | 19.95 ± 5.82 | 22.88 ± 7 | 24.54 ± 6.97 | ns | ns | ns |
| t30 | 18.32 ± 3.96 | 15.13 ± 2.88 | 14.91 ± 2.92 | ns | ns | ns |
| t60 | 18.81 ± 4.98 | 13.8 ± 3.07 | 13.98 ± 3.04 | ns | ns | ns |
| t120 | 23.53 ± 7.9 | 17.19 ± 3.95 | 17.57 ± 3.88 | ns | ns | ns |
| MMTT Glucose clearance (mM) |  |  |  |  |  |  |
| t0 | 8.04 ± 0.33 | 8.71 ± 0.41 | 8.61 ± 0.42 | ns | ns | ns |
| t5 | 10.64 ± 0.55 | 11.88 ± 0.47 | 11.95 ± 0.47 | ns | ns | ns |
| t10 | 14.89 ± 0.89 | 12.82 ± 0.86 | 12.73 ± 0.88 | ns | ns | ns |
| t20 | 11.61 ± 0.25 | 12.31 ± 1.04 | 12.73 ± 1.12 | ns | ns | ns |
| t30 | 11.48 ± 0.38 | 12.21 ± 0.72 | 12 ± 0.76 | ns | ns | ns |
| t45 | 10.88 ± 0.32 | 10.8 ± 0.69 | 10.81 ± 0.69 | ns | ns | ns |
| t60 | 10.51 ± 0.28 | 10.49 ± 0.32 | 10.5 ± 0.32 | ns | ns | ns |
| t90 | 9.41 ± 0.27 | 8.97 ± 0.23 | 9.02 ± 0.23 | ns | ns | ns |
| t120 | 9.33 ± 0.41 | 8.09 ± 0.26 | 8.11 ± 0.25 | ns | ns | ns |
